# Use of the Foot-and-Mouth Disease Virus 2A Peptide Co-Expression System to Study Intracellular Protein Trafficking in *Arabidopsis*

**DOI:** 10.1371/journal.pone.0051973

**Published:** 2012-12-14

**Authors:** Stefan Burén, Cristina Ortega-Villasante, Krisztina Ötvös, Göran Samuelsson, László Bakó, Arsenio Villarejo

**Affiliations:** 1 Umeå Plant Science Centre, Department of Plant Physiology, Umeå University, Umeå, Sweden; 2 Department of Biology, Universidad Autónoma de Madrid, Madrid, Spain; 3 Plant Biology, Biological Research Center, Hungarian Academy of Sciences, Szeged, Hungary; University of Nebraska Medical Center, United States of America

## Abstract

**Background:**

A tool for stoichiometric co-expression of effector and target proteins to study intracellular protein trafficking processes has been provided by the so called 2A peptide technology. In this system, the 16–20 amino acid 2A peptide from RNA viruses allows synthesis of multiple gene products from single transcripts. However, so far the use of the 2A technology in plant systems has been limited.

**Methodology/Principal Findings:**

The aim of this work was to assess the suitability of the 2A peptide technology to study the effects exerted by dominant mutant forms of three small GTPase proteins, RABD2a, SAR1, and ARF1 on intracellular protein trafficking in plant cells. Special emphasis was given to CAH1 protein from *Arabidopsis*, which is trafficking to the chloroplast via a poorly characterized endoplasmic reticulum-to-Golgi pathway. Dominant negative mutants for these GTPases were co-expressed with fluorescent marker proteins as polyproteins separated by a 20 residue self-cleaving 2A peptide. Cleavage efficiency analysis of the generated polyproteins showed that functionality of the 2A peptide was influenced by several factors. This enabled us to design constructs with greatly increased cleavage efficiency compared to previous studies. The dominant negative GTPase variants resulting from cleavage of these 2A peptide constructs were found to be stable and active, and were successfully used to study the inhibitory effect on trafficking of the N-glycosylated CAH1 protein through the endomembrane system.

**Conclusions/Significance:**

We demonstrate that the 2A peptide is a suitable tool when studying plant intracellular protein trafficking and that transient protoplast and *in planta* expression of mutant forms of SAR1 and RABD2a disrupts CAH1 trafficking. Similarly, expression of dominant ARF1 mutants also caused inhibition of CAH1 trafficking to a different extent. These results indicate that early trafficking of the plastid glycoprotein CAH1 depends on canonical vesicular transport mechanisms operating between the endoplasmic reticulum and Golgi apparatus.

## Introduction

The plant endomembrane system is a highly dynamic structure and comprises several biochemically distinct membrane-bound organelles that are linked by membrane traffic. Each protein entering the endomembrane system must be transported to its correct destination and each compartment has to maintain its distinct molecular identity. To accomplish this, efficient sorting mechanisms and targeting functions have to be maintained. Ras-like small GTP-binding proteins, such as SAR1, ARF1, and RAB GTPases, play an important role in the general mechanism of vesicular trafficking pathways in all eukaryotic cells [1], [2]. They share a common structure and act as molecular switches by cycling between active GTP-bound and inactive GDP-bound states [1]. The use of negative mutants that are locked at the GTP- or GDP-bound state, acting dominantly over the wild type proteins, has advanced the understanding of their function in plant intracellular vesicular trafficking [2], [3], [4]. Transient expression of such mutants has shown that SAR1 controls the assembly of the protein COPII coat that direct vesicle budding from endoplasmic reticulum (ER), whereas the ARF1 GTPase performs a similar function for Golgi-derived COPI vesicle budding as well as post-Golgi traffic [5], [6], [7]. Expression of a mutated form of RABD2a, containing a single amino acid substitution in the conserved GTP binding motif, showed to be a dominant inhibitor, and revealed its role in targeting and fusion of ER-derived COPII vesicles at the Golgi surface [8].

Recently, evidence for a chloroplast protein transport pathway involving the ER and Golgi apparatus in *Arabidopsis* has been presented [9], [10]. The carbonic anhydrase 1 (CAH1) protein was found to localize in the chloroplast stroma, despite its predicted ER signal peptide. Application of brefeldin A (BFA), a widely used fungal metabolite that interferes with Golgi-mediated vesicle traffic, obstructed transport of CAH1 to the chloroplast, causing it to arrest within the endomembrane system. The stromal protein was also shown to be N-glycosylated, confirming its transport via the endomembrane system to the chloroplast. Since then, other chloroplast proteins, including the rice α-amylase isoform I-1 (Amyl-1) and nucleotide pyrophosphatase/phosphodiesterase 1 (NPP1), were shown to follow the same or similar targeting pathways [11], [12], [13], indicating that several proteins might be transported to chloroplasts involving this pathway.

Although no direct experimental evidence for the mechanism whereby the above mentioned plastid proteins are transported from the Golgi apparatus to the plastids has been presented, trafficking from the ER to the Golgi, at least in monocot species, seems to depend on canonical elements such as ARF1 and SAR1 [13]. The incorporation of Golgi-resident proteins into plastids in both rice and onion cells appeared to be stimulated by expression of Amyl-1 [13]. These data suggest that communication between these compartments might be tightly regulated *in vivo* and that fine tuned expression of elements involved in vesicular trafficking and plastid N-glycoproteins must occur.

While the *Arabidopsis* CAH1 protein harbours complex type *N*-glycans typical for proteins trafficking through the Golgi [9], [14], the rice NPP1 and Amyl-1 seem to be modified with high-mannose type *N*-glycans characteristic for the ER [11], [12]. Whether these differences reflect species-specific transport mechanisms remains to be clarified. Therefore, a molecular and genetic dissection of the elements involved in trafficking of these plastid glycoproteins is of great importance for our understanding of intracellular plant cell communication.

To study the effect of dominant inhibitory GTPases on CAH1 trafficking, while avoiding secondary effects and lethality of the plant cells, we aimed to develop an experimental system for transient co-expression of such mutant proteins with CAH1.

**Figure 1:**
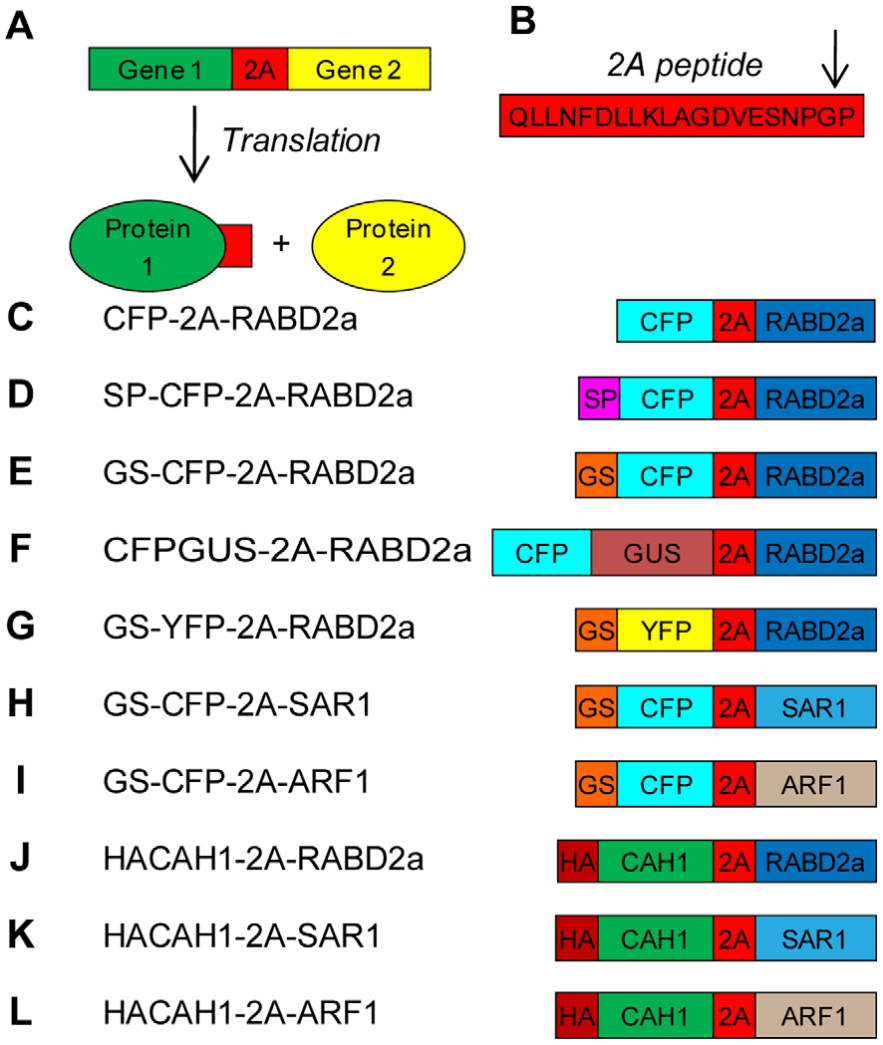
Schematic representation of 2A function and constructs. (A) Two individual polypeptides can be generated from one transcript using 2A to link the individual genes. (B) 2A peptide sequence and its cleavage site (G/P). (C–L) The constructs used in this paper are described in the following scheme: 2A, 20 amino acid 2A peptide; CFP, enhanced cyan fluorescent protein; YFP, enhanced yellow fluorescent protein; SP, ER signal peptide of CAH1 protein; GS, Golgi targeting signal of N-acetylglucosaminyl transferase I; GUS, β-glucuronidase; RABD2a, *Arabidopsis* RABD2a GTPase; SAR1, *Nicotiana tabacum* SAR1p; ARF1, *Arabidopsis* ADP-ribosylation factor 1; HA, hemagglutinin epitope tag; CAH1, *Arabidopsis* α-CAH1. All GTPases were cloned as wild type and dominant mutant versions.

Transient expression techniques, such as protoplast transfection, usually results in a heterogeneous population of transfected/non-transfected cells. While transfection efficiency can vary from relatively low to significant, non-transfected cells will always be present and reduce or mask the effect on the total population as such. To circumvent this problem, fluorescent marker proteins are often fused to the protein of interest in order to enable visualization and analysis of the transfected cells. Unfortunately, previous studies on GTPases indicate that these proteins are sensitive to modifications, resulting in unstable forms with no activity when tagged at the N-terminus, and stable but with decreased activity when tagged at the C-terminus [15]. One solution could be the use of the 2A peptide technology. The 2A peptide (hereafter called 2A) is a 16–20 amino acid long peptide used by some RNA viruses for synthesis of multiple gene products (proteins) from single transcripts [16], [17], [18]. During translation of the polyprotein gene, 2A causes a premature release of the polypeptide amino (N)-terminal of 2A, without requirement of extra-ribosomal factors or interfering with subsequent translation reaction (Figure 1A). Although use of 2A technology has previously been reported in plants [16], [18], [19], Samalova and co-workers [15] have questioned its use in plant systems, demonstrating that further development and characterization of the 2A system is necessary.

In this study, we wanted to improve the 2A technology for use in plant cells as a tool to study basic cellular processes, such as protein trafficking mechanisms. After successful optimization of 2A cleavage efficiency, mutant forms of small GTP-binding proteins could be expressed and used to interfere with trafficking of CAH1 at the endomembrane system. The results presented in this work highlight the 2A technology as a valuable tool for effective and stoichiometric co-expression of marker and effector molecules in plant systems. In addition, our study demonstrates that 2A mediated transient co-expression of fluorescent markers combined with fluorescent activated cell sorting can be used to obtain homogeneous mutant protoplast populations in very short time.

## Results

### Strategy of Polyprotein Construction and Expression

To test and improve the 2A technology for use in plant cells, the effect of sequence and subcellular targeting on 2A cleavage efficiency was tested. A panel of 2A polyprotein constructs were generated (Figure 1C–L) using the coding sequences for different genes or gene fragments, such as enhanced cyan/yellow fluorescent protein (CFP/YFP), β-glucuronidase (GUS), Golgi targeting signal from N-acetylglucosaminyl transferase I (GS) [20], ER targeting signal peptide (SP) from CAH1 [9], RAB GTPase D2a (RABD2a, At1g02130) [8], [21], secretion associated, ras-related protein1 (SAR1, accession AF210431) [22], ADP-ribosylation factor 1 (ARF1, At2g47170) [23] and N-terminally HA epitope-tagged CAH1 (HACAH1) [24]. For each construct, the gene fragments were separated by the 19 C-terminal amino acid sequence of 2A together with the N-terminal 2B proline [25]. These 20 amino acids are collectively called 2A from here on (Figure 1). All constructs were controlled by one or two copies of the constitutively active Cauliflower mosaic virus 35S promoter for transient expression in transfected *Arabidopsis* protoplasts or *Agrobacterium* transformed *Nicotiana benthamiana* leaves. CFP *N*-terminal of 2A allowed visualization of transfected cells using fluorescence microscopy and anti-GFP antibodies, cross-reacting with CFP, facilitated immunoblot-based estimation of cleavage efficiency for the different constructs.

### 2A Mediated Polyprotein Cleavage Resulted in Anticipated Localization of the Released Proteins

**Figure 2:**
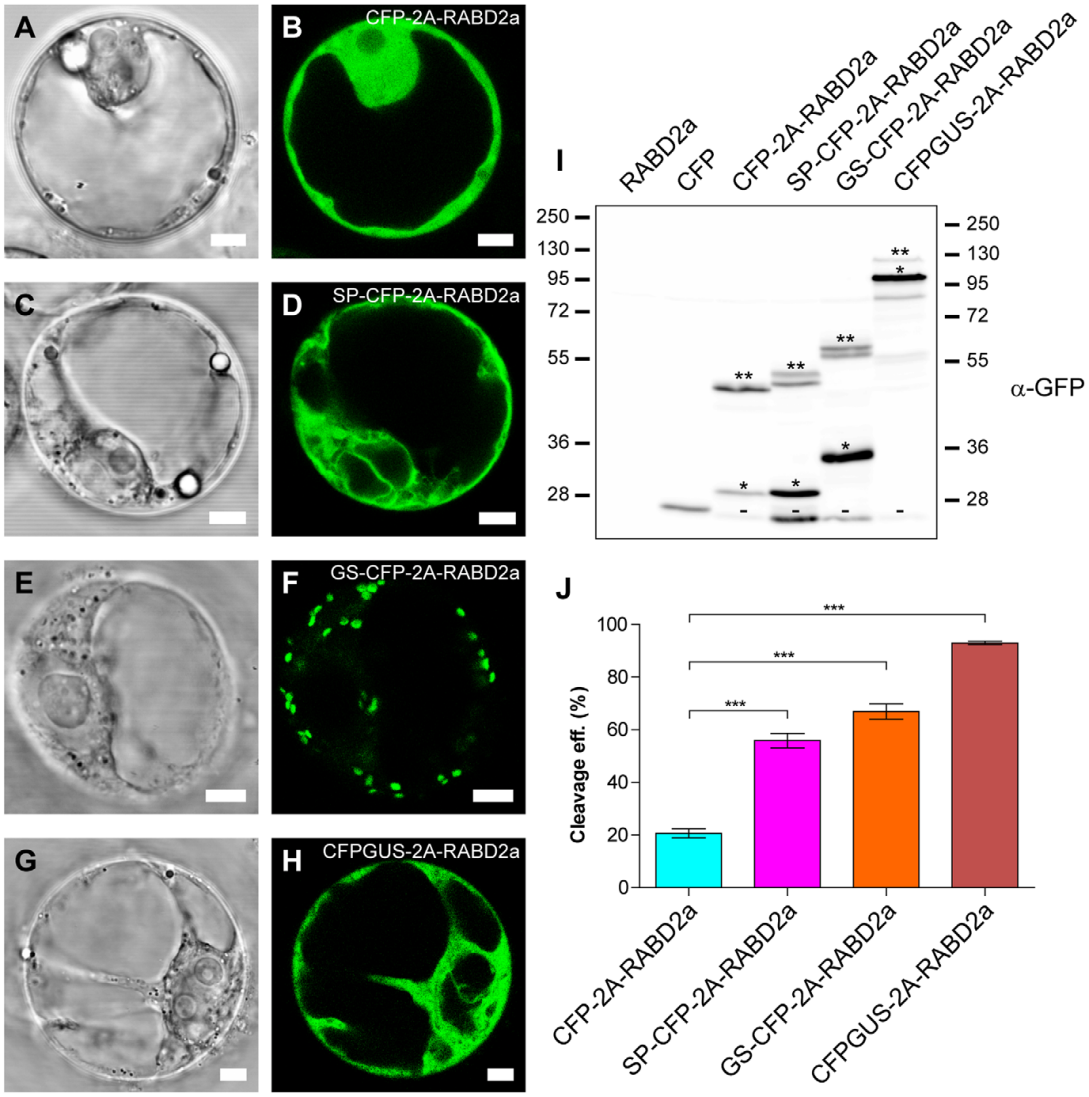
Targeting of 2A constructs and 2A cleavage efficiency. (A–H) Bright field and confocal images of *Arabidopsis* protoplasts transiently expressing CFP-2A-RABD2a (A and B), SP-CFP-2A-RABD2a (C and D), GS-CFP-2A-RABD2a (E and F) and CFPGUS-2A-RABD2a (G and H). Bars = 5 µm. (I) Immunoblot analysis of protein extracts from *Arabidopsis* protoplasts transiently transfected with non-tagged RABD2a (negative control), CFP (positive control), CFP-2A-RABD2a, SP-CFP-2A-RABD2a, GS-CFP-2A-RABD2a and CFPGUS-2A-RABD2a using anti-GFP antiserum. 2A cleaved (*), non-cleaved full-length 2A polyproteins (**) and putative degradation products (−) are indicated. Protein loading was adjusted in order to highlight both cleaved and non-cleaved product for each construct. (J) Cleavage efficiency of the different 2A constructs was estimated from the amounts of cleaved (*) versus non-cleaved (**) products as described in *Material and Methods*. Error bars show standard error (n = 4, *** = p<0.001).

Wild type and dominant mutant derivatives of three small GTPases; RABD2a, SAR1, and ARF1 [1], [5], [23], [26] were fused to the carboxyl (C)-terminus of 2A. Confocal laser scanning microscopy of protoplasts transfected with the various 2A wild type constructs revealed the expected CFP localization pattern (Figure 2A–H). Soluble constructs lacking targeting information, CFP-2A-RABD2a and CFPGUS-2A-RABD2a, localized to the cytosol (Figure 2A–B, G–H). CFP fluorescence from CFP-2A-RABD2a could also be seen in the nucleus (Figure S1B), since the 29 kDa CFP-2A product can enter through the nuclear pore [27]. The two constructs with N-terminal extensions, the signal peptide of CAH1 (SP) [9], (Figure 1D and Figure 2C–D), or the N-terminal part of N-acetylglucosaminyl transferase I (GS) [20], (Figure 1E and Figure 2E–F) are both translated by ER bound ribosomes [1]. These constructs revealed structures typical of endomembrane appearance, such as reticulated ER-like fluorescence for the SP-CFP-2A construct *en route* to the apoplast (Figure 2C–D), and dot-like structures for the Golgi-targeted GS-2A construct (Figure 2E–F). Correct subcellular targeting and localization of 2A tagged marker protein was further confirmed by co-localization with established markers (Figure S1). As expected, addition of 2A seemed not to cause mistargeting or affect localization of the marker protein, as seen from co-localization of GS-CFP and GS-YFP-2A-RABD2A (Figure S2) [7].

### Subcellular Targeting of 2A Constructs Affects the Cleavage Efficiency of the Polyprotein

To test the effect of subcellular targeting on cleavage efficiency of 2A, three different constructs were studied according to Figure 1C–E. All constructs shared a common core consisting of CFP-2A-RABD2a. Expression of the cytosolically translated CFP-2A-RABD2a resulted in rather low cleavage efficiency (Figure 2I (lane 3) and 2J), about 20% and consistent with previous reports [15]. Interestingly, targeting of the construct to the ER greatly increased the cleavage efficiency to about 60% (Figure 2I (lane 4) and 2J). Similar cleavage efficiency was observed for the Golgi targeted construct (Figure 2I (lane 5) and 2J). Two bands of non-cleaved, full-length, fusion protein could be detected for SP-CFP-2A-RABD2a and GS-CFP-2A-RABD2a (Figure 2I (lane 4 and 5), bands indicated with **), where the higher molecular mass isoforms presumably corresponded to fusion proteins where the signal peptides had not (yet) been processed. An additional band at around 28 kDa cross-reacting with the GFP antibodies could also be seen in all 2A extracts (Figure 2I, bands indicated with -). Intriguingly, these bands showed similar migration patterns despite of the difference in size between the expressed 2A constructs, suggesting that they originated from degradation products of the CFP moieties.

### Insertion of GUS N-terminal to the 2A Sequence Increases Cleavage Efficiency of the Cytosolically Expressed Construct

The results presented above raised the question whether improved cleavage efficiency of 2A was solely due to translation by ER-membrane-bound ribosomes. To test this hypothesis, a new construct was generated in which GUS was inserted between CFP and 2A (Figures 1F and 2G and 2H). Cleavage efficiency for the resulting 2A polyprotein was almost 95% (Figure 2I (lane 6), 2J and Figure S3), indicating that cleavage efficiency not only depends on subcellular targeting, but also on the sequence N-terminal of 2A and that functionality of 2A needs to be verified for each case.

### High Cleavage Efficiency Results in Increased Levels of Stable RABD2a Protein

**Figure 3:**
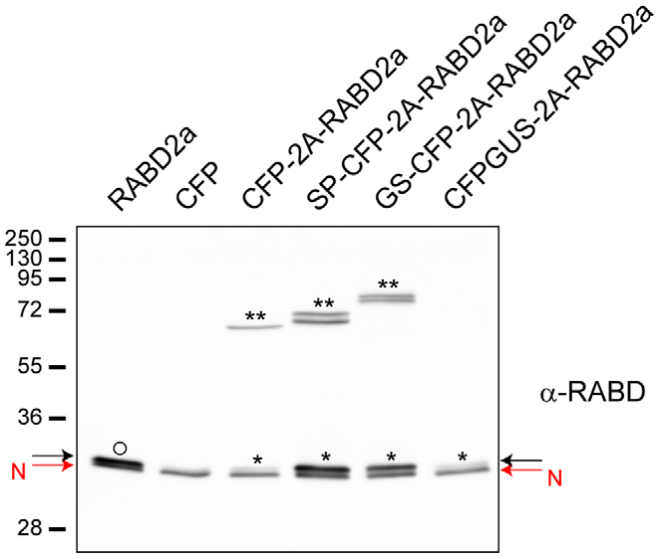
RABD2a protein is stable when expressed from 2A constructs. Immunoblot analysis of protein extracts from *Arabidopsis* protoplasts transiently transfected with non-tagged RABD2a (positive control), CFP (negative control), CFP-2A-RABD2a, SP-CFP-2A-RABD2a, GS-CFP-2A-RABD2a and CFPGUS-2A-RABD2a using anti-RABD2 antiserum. Native RABD (red arrow, N), overexpressed RABD2a (black arrow, O), 2A derived RABD2a (black arrow, *) and non-cleaved 2A polyprotein (**) are indicated. Low expression and high cleavage efficiency of CFPGUS-2A-RABD2a construct makes no detectable full length CFPGUS-2A-RABD2a band, and only faint band of released RABD2a (*) visible at this exposure time.

A previous study reported that RABD2a originating from 2A constructs exhibits low activity or is unstable [15]. To address the issue of stability of 2A generated RABD2a, protoplasts were transfected with the different RABD2a-expressing constructs (Figure 1C–F). Corresponding protein extracts were analyzed by Western blotting using RABD antibodies (kindly provided by Dr. Ian Moore, Oxford). The ratio of released RABD2a to non-cleaved polyprotein was similar to what was seen from analysis of the CFP moiety using GFP antibodies (Figure 2I and Figure 3), suggesting that the residual proline at the N-terminus of RABD2a was not affecting the stability of the protein. In addition, the 2A derived RABD2a was migrating at the same molecular mass as over-expressed untagged RABD2a, slightly higher than the native isoform detected by the RABD antibody (Figure 3, black and red arrows respectively) (Dr. Ian Moore, Oxford, personal communication). This verified that 2A cleavage indeed was specific, and that low levels of full length polyproteins were not due to premature termination of transcription or translation.

Stability of the different constructs was further assessed by cycloheximide treatment (CHX) (Figure S4). Protoplasts in which protein biosynthesis was inhibited by the drug showed no significant difference in cleavage efficiency ratio within the time frame of the experiment (up to 6 h). This confirms that the cleavage efficiency estimated in Figure 2I and 2J for the different 2A-peptide constructs was correct and not because of secondary effects or different susceptibility of the polyproteins to degradation [15].

### 2A-derived Dominant-negative Small GTPases are Effective after 2A Cleavage

An important issue regarding application of the 2A technology in the study of small GTPases (such as RABD2a, SAR1 and ARF1) [1], [6] is not only stability, but also the functionality of the protein released from the 2A polyprotein. Therefore we tested the effect of dominant negative RABD2a version (RABD2a (N121I)) [8], GTP-locked SAR1 (SAR1 (H74L)) [5], [28] and GDP- and GTP-locked versions of ARF1 (ARF1 (T31N) and ARF1 (Q71L), respectively) [7], [23] on secretion of SP-CFP-2A to the apoplast and targeting of GS-CFP-2A to the Golgi.

**Figure 4:**
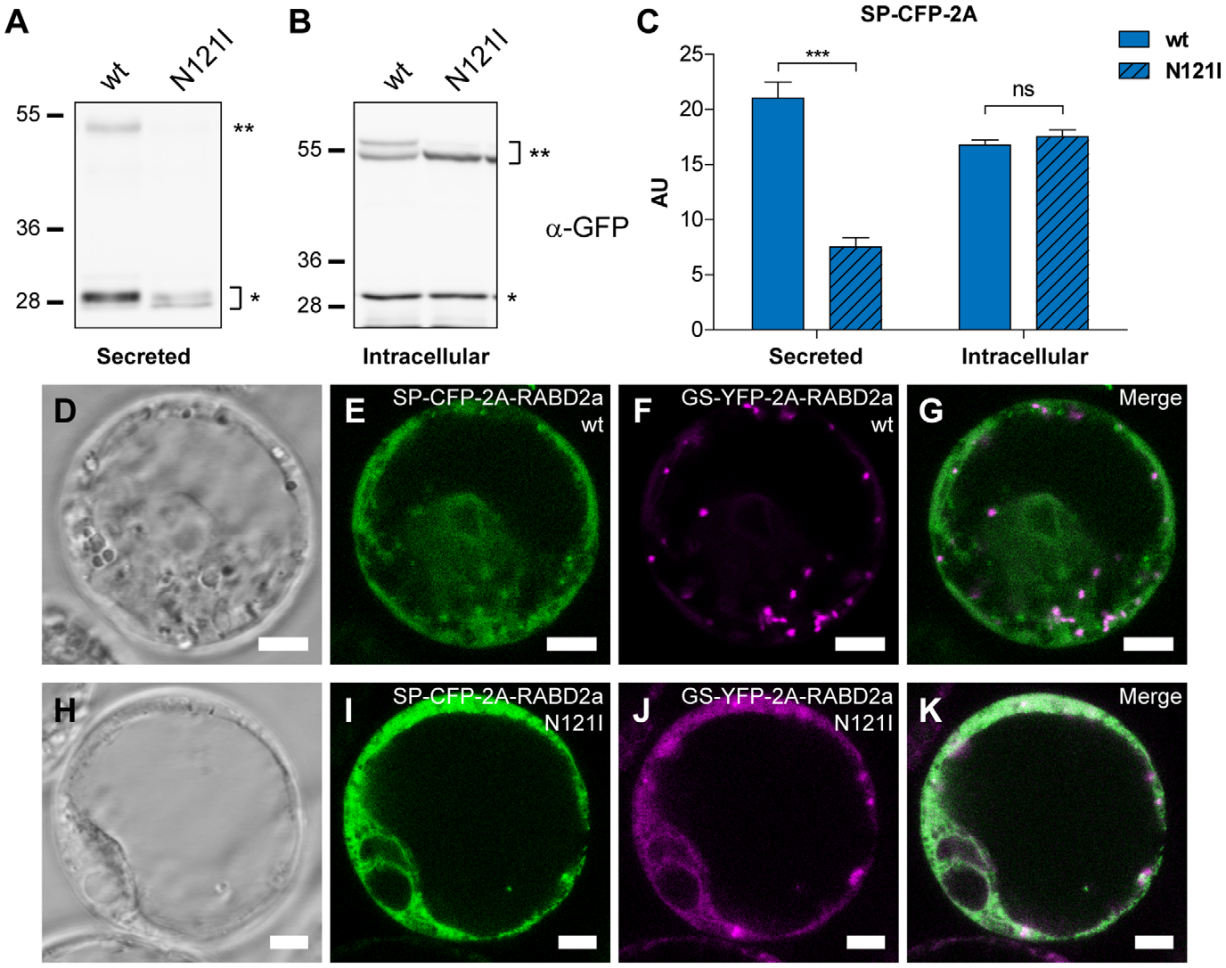
Analysis of activity of the RABD2a protein originating from 2A constructs. (A and B) Immunoblot analysis using anti-GFP antiserum of secreted (A) and intracellular (B) protein extracts from *Arabidopsis* protoplasts transiently transfected with (wt) or mutant (N121I) SP-CFP-2A-RABD2a. Upper band (**) corresponds to non-cleaved full length SP-CFP-2A-RABD2a and lower (*) to cleaved SP-CFP-2A polypeptide. (C) The amount of secreted (Secreted) and non-secreted intracellular SP-CFP-2A (Intracellular) were quantified. AU, arbitrary unit, error bars show standard error (n = 4, *** = p<0.001). (D-K) Confocal images of transiently transfected *Arabidopsis* protoplasts co-expressing wild type (D-G) or mutant (N121I) forms (H-K) of SP-CFP-2A-RABD2a and GS-YFP-2A-RABD2a. CFP channel is shown in green and YFP channel in magenta. Merged images (G and K) show co-localization (white). Bars = 5 µm.

As shown in Figure 4A (left lane), when protoplasts were transfected with SP-CFP-2A-RABD2a (wt), cleaved SP-CFP-2A (bands indicated with *) and small amounts of non-cleaved SP-CFP-2A-RABD2a (band indicated with **) were detected in the apoplastic protein extract. However, expression of the RABD2a (N121I) mutant construct (Figure 4A, right lane) strongly reduced secretion of SP-CFP-2A into the media (Figure 4C). By contrast, the levels of intracellular SP-CFP-2A and SP-CFP-2A-RABD2a were similar for both constructs (Figure 4B and 4C), indicating that impaired secretion was caused by inhibited protein trafficking by RABD2a (N121I) form and not a result of reduced expression or protein stability.

To further confirm activity of 2A-derived RABD2a protein, protoplasts were co-transfected with GS-YFP-2A-RABD2a and SP-CFP-2A-RABD2a. Protoplasts transfected with wild type GS-YFP-2A-RABD2a and SP-CFP-2A-RABD2a (Figure 4D–G) showed YFP fluorescence confined to numerous mobile punctuate structures typical of Golgi stacks (Figure 4F) and reticulated CFP fluorescence (Figure 4E). On the contrary, co-expression of mutant isoforms resulted in increased intracellular CFP fluorescence, together with a dramatically altered sub-cellular distribution of YFP in an ER-like reticulated network mainly co-localizing with SP-CFP-2A (Figure 4H–K). As expected, estimation of the correlation for SP-CFP/GS-YFP signals using Pearson’s coefficient [29] showed a weak co-localization when co-expressed together with wild type version of RABD2a (0.46±0.13, average±standard deviation). This low level of co-localization possibly corresponded to GS-YFP-2A on its way to the Golgi and secreted SP-CFP-2A transiently passing the Golgi apparatus in the way to the plasma membrane. Importantly, expression of the RABD2a (N121I) mutant isoform significantly increased co-localization of GS-YFP-2A and SP-CFP-2A (Figure 4K) (0.74±0.10, average±standard deviation, p<0.001), indicating that GS-YFP-2A was retained in ER-like structures together with SP-CFP-2A. These data confirmed that RABD2a released from 2A constructs was stable and active, and that the mutant form inhibited trafficking of proteins from the ER to the Golgi apparatus.

### Presence of Degradation Products Might Indicate Vacuolar Mistargeting

Western blot analysis of protein extracts using GFP antibodies revealed a 28 kDa product (Figure 2I, bands indicated with -), indicating that part of the non-cleaved 2A polypeptide, or the CFP-2A product itself, was further processed or partly degraded. The low molecular mass CFP variant was more abundant in the constructs translated by ER-bound ribosomes (SP-CFP-2A and GS-CFP-2A). Previous studies have suggested that 2A could act as a vacuolar targeting signal in plant cells [15]. In fact, the polypeptide migrating around 28 kDa strongly resembled the product that was detected following vacuolar degradation of GFP [30], corroborating that some of the CFP-2A containing polypeptide likely was mistargeted to the vacuole. To further elaborate this observation, two additional constructs were expressed in protoplasts, one in which the RABD2a moiety was removed (GS-CFP-2A) and another harbouring only GS-CFP (Figure S5A and S5B). Surprisingly, a product with similar migration could be detected in protein extracts from all three constructs, indicating that the 28 kDa product was not unique or due to the 2A sequence itself (Figure S5C, bands indicated with -). Interestingly, BFA treatment of protoplasts expressing wild type GS-CFP-2A-RABD2a resulted in reduced levels of the presumable 28 kDa degradation product (Figure S6A, lane 2 vs. 1, bands indicated with -), suggesting that inhibited membrane trafficking also reduced protein processing. The same effect was seen when co-expressed with mutant RABD2a (N121I) (Figure S6A, lane 3 vs. 1, bands indicated with -). Quantification of the ratio of processed to non-processed GS-CFP-2A (Figure S6A, bands indicated with - and *, respectively) showed that this effect was similar or even more pronounced when GS-CFP-2A was expressed with RABD2a (N121I), as compared to wild type RABD2a in the presence of BFA (Figure S6B). Reduced levels of degradation product detected in cells incubated with BFA or expressing RABD2a (N121I) suggested that this action was occurring in the vacuole, as previously reported for GFP polypeptides [30], and confirming that 2A-derived RABD2a (N121I) was inhibiting protein trafficking.

### 2A can be Used as a Universal Experimental System for Co-expression of Trafficking Elements other than RABD2a Protein

**Figure 5:**
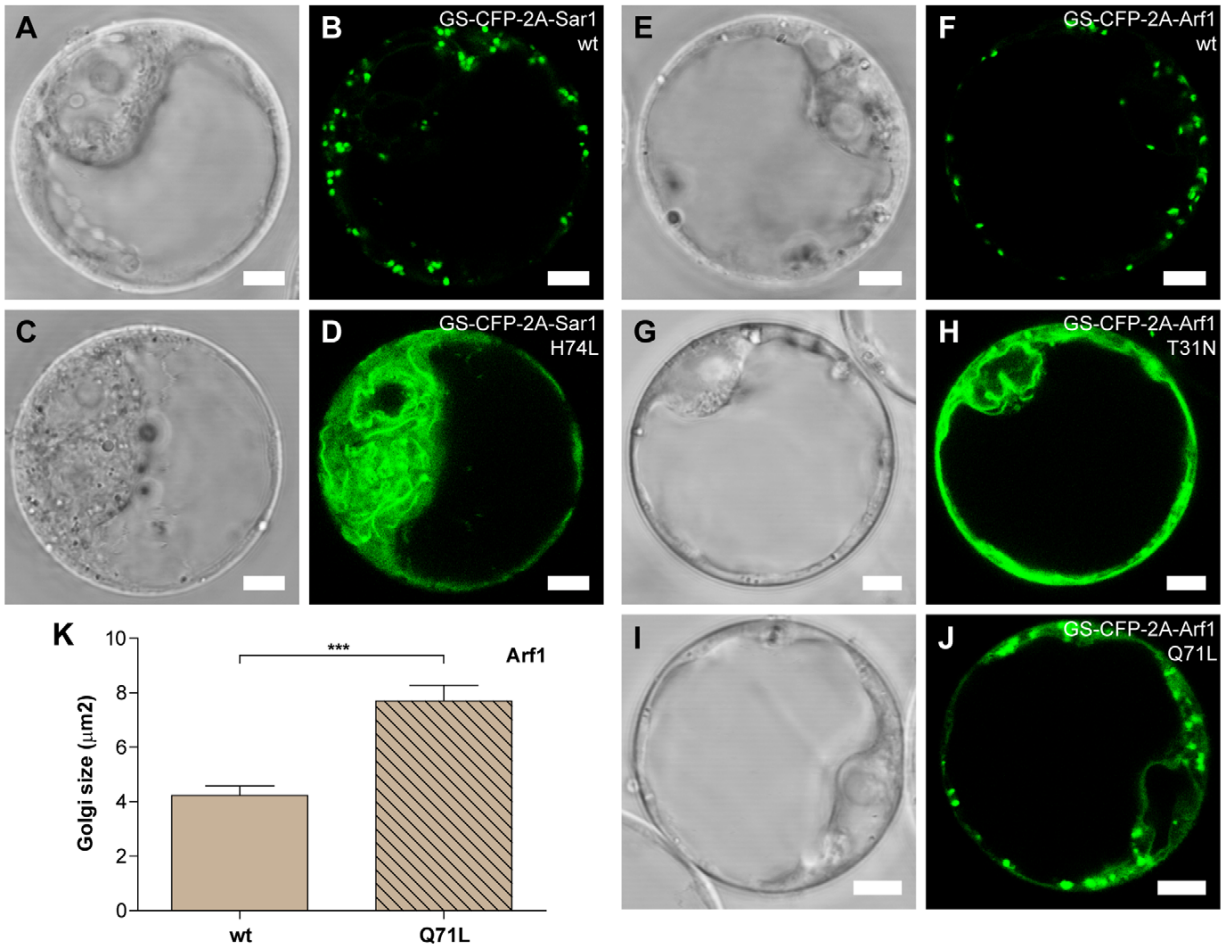
Analysis of activity of SAR1 and ARF1originating from 2A constructs. Representative confocal images of *Arabidopsis* protoplasts transiently expressing GS-CFP-2A-SAR1 (wt) (A and B), GS-CFP-2A-SAR1 (H74L) (C and D); GS-CFP-2A-ARF1 (wt) (E and F), GS-CFP-2A-ARF1 (T31N) (G and H) and GS-CFP-2A-ARF1 (Q71L) (I and J). Bars = 5 µm. (K) Analysis of fluorescent Golgi stacks size in *Arabidopsis* protoplasts transiently expressing GS-CFP-2A-ARF1 (wt) or mutant (Q71L) forms. Error bars show standard error (n = 14, *** = p<0.001).

Two other small GTPases frequently studied for their involvement in protein trafficking are SAR1 and ARF1 [6]. SAR1 (H74L) has been shown to cause a fluorescent Golgi marker to accumulate in the ER [3], [21], [28], while ARF1 (T31N) and ARF1 (Q71L) appear to affect marker proteins in slightly different ways [4], [23]. To demonstrate that 2A can be used for stoichiometric co-expression of active SAR1 and ARF1 polypeptides, the effect of these mutant isoforms was tested. SAR1 (H74L) and ARF1 (T31N and Q71L) were cloned C-terminally of 2A containing Golgi marker GS-CFP (Figure 1H and 1I). Co-expression of the mutant GS-CFP-2A-SAR1 (H74L) strongly affected localization of the cleaved GS-CFP-2A marker, changing its punctuate Golgi labelling to a reticulated, ER-like, structure (Figure 5A–D) as previously reported for other Golgi markers [3], [21], [28]. In a similar way, ARF1 (T31N) caused GS-CFP-2A to accumulate in ER-like structures (Figure 5E–H), as previously shown for the sialyltransferase fused to GFP (*N*-ST-GFP) [23]. In addition, a subpopulation of protoplasts showed structures resembling vacuolar compartments (Figure S7), presumably indicating mistargeting of the GS-CFP-2A construct in some ARF1 (T31N) expressing cells. On the contrary, co-expression of ARF1 (Q71L) did not result in clear ER-like fluorescence. Reticulate labelling was increased, but most of the fluorescence remained in Golgi-like structures (Figure 5I–J). The appearance of the Golgi seemed to be altered though, with enlarged GS-CFP-2A fluorescing structures compared to protoplasts co-expressing the wild type ARF1 protein (Figure 5J and 5F). Estimation of the size of these fluorescent dot-like structures confirmed this observation (Figure 5K).

Our data shows that similar to RABD2a (N121I), dominant mutant versions of SAR1 and ARF1 originating from the GS-CFP-2A construct were functional and gave a phenotypic effect on the Golgi-localized marker protein. The effects were consistent to those observed in previous studies [4], [7], [23]. Our findings clearly demonstrate the value of a stoichiometric co-expression system that can easily be applied to live-cell imaging and confocal microscopy studies. These results prompted us to further extend and test the versatility of the 2A system in combination with other experimental techniques, such as biochemical assays and fluorescence-activated cell sorting.

### Application of the 2A Technology in a Rapid and Easy Biochemical Assay to Reveal the Effect of Small GTPases on Trafficking of CAH1: Inhibition of Anterograde and Retrograde ER-Golgi Vesicle Transport Affects CAH1

To extend our understanding of the pathway followed by the *Arabidopsis* α-type CAH1 through the ER and Golgi [9], and to test whether the 2A co-expression system could be combined with a rapid biochemical assay to dissect the trafficking route employed by this protein, HA-epitope tagged CAH1 (HACAH1, [24]) was co-expressed with dominant inhibitory variants of these GTPases in *Arabidopsis* protoplasts (Figure 1J–L). This experimental arrangement ensures that HACAH1 would always be expressed at the same time and at similar levels as the GTPase studied. Immunogold labelling experiments revealed that this construct is mainly targeted to the chloroplast in Col-0 *Arabidopsis thaliana* suspension-cultured cells used for protoplast transfection experiments, though a small portion of the labelling was also observed over the endomembrane system (Table S1), as shown in other *Arabidopsis thaliana* suspension-cultured cells [24].

Resistance or susceptibility to endo-β-*N*-acetylglucosaminidase H (Endo H), an enzyme able to deglycosylate high mannose type protein *N*-linked glycans, i.e. proteins containing glycans that have not been processed by glycosidases and glycosyltransferases in the Golgi, can be used as a method to assess CAH1 trafficking through the endomembrane system [8]. This is based on the fact that CAH1 has five potential *N*-glycosylation sites, which have been shown to be occupied at least partly with complex type *N*-glycans [9], [24]. Fractionation of transiently transformed *Nicotiana benthamiana* leaves expressing wild type HACAH1-2A-RABD2a showed that HACAH1-2A present in the soluble stroma-containing fraction was mostly resistant to Endo H treatment, while HACAH1-2A originating from the microsome and ER-containing fraction was mainly sensitive to Endo H (Figure S8). This result encouraged us to use Endo H susceptibility as a biochemical assay to study the effect of the different dominant mutant GTPases on endomembrane trafficking of HA-tagged CAH1, as inhibited trafficking from the ER would make the HA-CAH1 glycans more susceptible to Endo H treatment.

**Figure 6:**
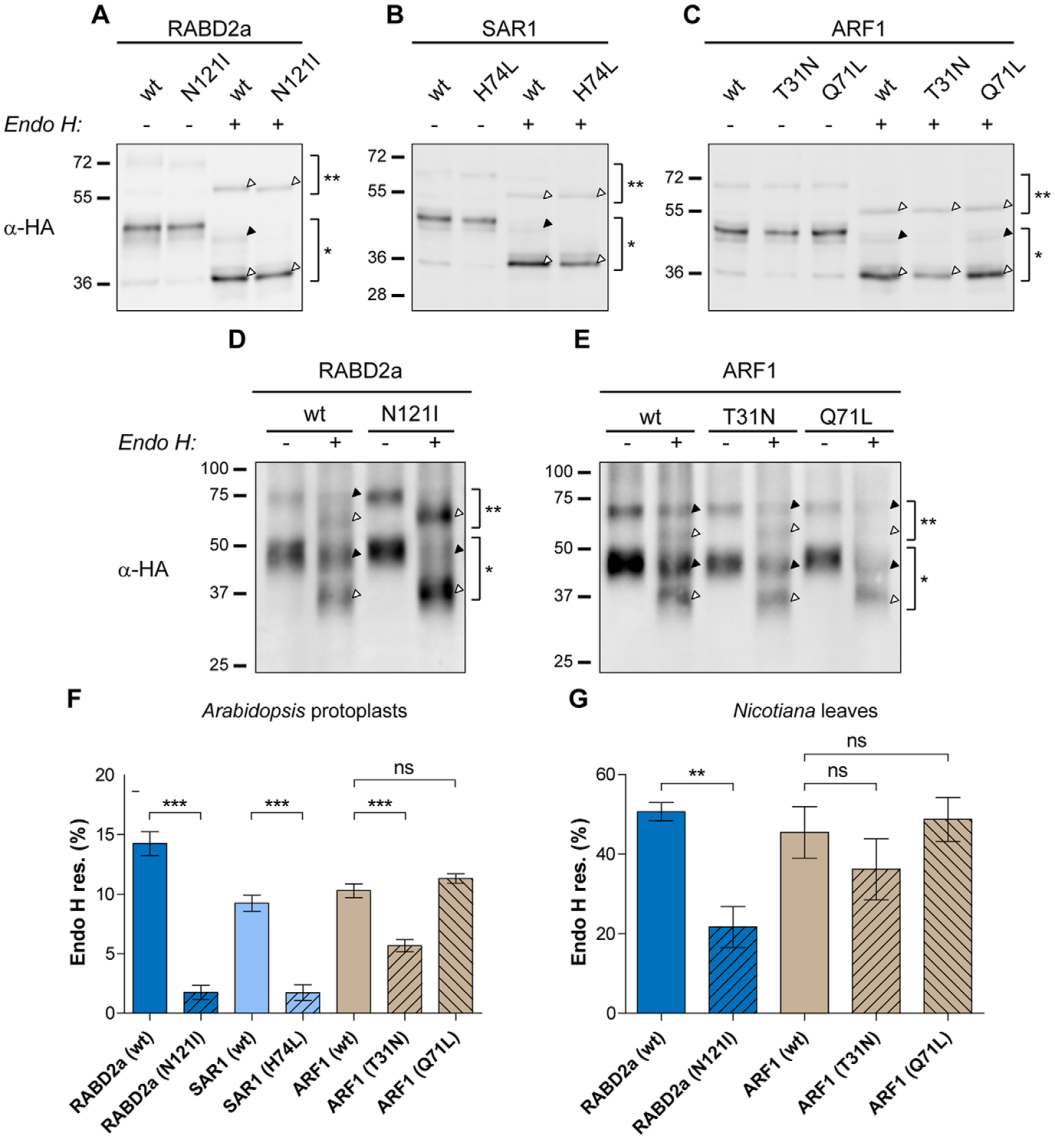
Effect of mutant forms of small GTPases on trafficking of HACAH1-2A. Immunoblot analysis using anti-HA antiserum of protein extracts from *Arabidopsis* protoplasts (A, B and C) and *Nicotiana benthamiana* leaves (D and E) expressing HACAH1-2A-RABD2a (wt) or mutant (N121I) form (A and D); HACAH1-2A-SAR1 (wt) or mutant (H74L) form (B); HACAH1-2A-ARF1 (wt) or mutant (T31N) and (Q71L) forms (C and E). Full length non-cleaved HACAH1-2A-RABD2A/SAR1/ARF1 (**), 2A cleaved HACAH1-2A (*), Endo H resistant (black triangle) and susceptible (white triangle) forms of HACAH1-2A are indicated. (F and G) The ratio of resistant (black triangle) to total HACAH1-2A (white+black triangle) cleaved forms (*) was calculated for Arabidopsis protoplasts (F) and *N. benthamiana* leaves (G). Error bars show standard error (n = 4, ** = p<0.01, *** = p<0.001).

Western blot analysis of protoplast extracts using anti-HA antibodies showed that the cleavage efficiency of HACAH1-2A-RABD2a/SAR1/ARF1 was similar or even higher to that observed for the fluorescent markers SP-CFP-2A and GS-CFP-2A (87.7 for HACAH1-2A-RABD2a; 89.2 for HACAH1-2A-ARF1; and 90.1 for HACAH1-2A-SAR1), confirming high cleavage efficiency for ER-targeted 2A constructs (Figure 6A, 6B and 6C, bands indicated with * and **, respectively). Immunoblot analysis further revealed the occurrence of several bands of slightly different molecular mass. Each of these bands could correspond to one specific glyco-isoform of the HACAH1-2A moiety, as seen for HA-CAH1 [24].

We observed that the non-cleaved HACAH1-2A-containing polyproteins were deglycosylated using Endo H, indicating that the non-cleaved variants were not trafficking further than the ER (Figure 6A, 6B and 6C, bands indicated with **, white arrow). Enzyme treatment of HACAH1-2A co-expressed with RABD2a (N121I) and SAR1 (H74L) showed a considerably higher portion of HACAH1-2A susceptible to Endo H, compared to HACAH1-2A co-expressed with the respective wild type GTPase (Figure 6A and 6B, bands indicated with *, black and white arrows). To quantify the difference in Endo H susceptibility of the wild type and mutant variants, the ratio of resistant to total HACAH1-2A was calculated. The ratio confirmed a significant effect when HACAH1-2A was co-expressed with dominant-mutant variants of these two GTPases, indicating the involvement of RABD2a and SAR1 in trafficking of HACAH1 between the ER and Golgi (Figure 6F). On the other hand, inhibition of retrograde transport between the Golgi and the ER has also been shown to block proteins destined to the vacuole and the plasma membrane [4], [23]. Blocked anterograde transport due to dominant mutant proteins responsible for retrograde transport is suggested to occur when the factors needed for the ER to Golgi transport cannot be recycled from the Golgi [2]. Endo H treatment of protein extracts from transfected protoplasts showed that HACAH1-2A accumulated as Endo H susceptible glycoforms when co-expressed with the GDP-locked ARF1 (T31N) mutant, similar to when being co-expressed with RABD2a (N121I) and SAR1 (H74L) (Figure 6C and 6F), suggesting that the protein was not transported further than the ER. This is in agreement with the effect of the ARF1 (T31N) mutant protein on the localization of GS-CFP-2A (Figure 5H). Interestingly the GTP-locked ARF1 (Q71L) mutant did not affect the susceptibility to Endo H (Figure 6C and 6F). Also the effect on the Golgi marker used in this study was weaker (Figure 5J), possibly indicating a lower inhibitory effect of the Q71L mutation in our expression system.

To demonstrate that the effect of mutant RABD2a, SAR1 and ARF1 on HACAH1 was not limited to protoplasts, transient co-expression of HACAH1-2A-RABD2a/SAR1/ARF1 in *Nicotiana benthamiana* leaves was achieved by *Agrobacterium* infiltration. Biochemical Endo H analysis of HACAH1-2A from leaf extracts revealed similar results *in planta* upon co-expression of mutant RABD2a and ARF1, as compared to transiently transfected protoplasts (Figure 6D, 6E and 6G). Cleavage efficiency was similar as for transient expression in protoplasts, 80±7% and 82±5% (±Standard deviation, n = 6) for HACAH1-2A-RABD2a and HACAH1-2A-ARF1, respectively, indicating that 2A cleavage is efficient in both experimental systems. The effect of ARF1 (T31N) appeared slightly weaker than observed in the protoplast system, and was not statistically significant in this study. The expression of HACAH1-2A-SAR1 constructs was for unknown reason very weak, especially for the mutant version, making Endo H assay difficult (data not shown).

Co-expression of HA-tagged CAH1 with dominant mutant GTPases involved in ER-to-Golgi trafficking not only demonstrated that the 2A system makes subsequent biochemical assays possible, but also confirmed the involvement of canonical elements in trafficking of CAH1 between the ER and the Golgi.

## Discussion

To continue our study of the mechanisms involved in transport of CAH1 between the ER and the Golgi, we explored the requirement for specific GTPases involved in vesicle trafficking. Although BFA arrested CAH1-GFP in aggregate-like structures [9], it is known to have different effects in different species, and even in different tissues within a species [31]. In addition, high BFA concentrations likely induce secondary effects, alterations that can be reduced using genetic tools.

The potential involvement of three individual GTPases was tested in this initial study; RABD2a, SAR1, and ARF1, all of them important for vesicle formation or docking at the ER-Golgi interface. Specific single point mutations in these GTPases can generate arrested and non-functional enzymes. RABD2a is a small GTPase involved in targeting and fusion of ER-derived COPII vesicles at the Golgi surface. Dominant negative variants where an N121I substitution was introduced in the GTP binding motif (RABD2a (N121I)), were shown to inhibit trafficking of secreted and Golgi targeted proteins out from the ER [1], [8], [32]. On the contrary, SAR1 and ARF1 are directly involved in the formation of COPII and COPI vesicles, respectively [1], [4]. Two mutant isoforms of ARF1 were tested: ARF1 (T31N) and ARF1 (Q71L), both affecting ER-to-Golgi trafficking and relocating Golgi markers to the ER [4]. The ARF1 (Q71L) mutant shows reduced GTPase activity, therefore acting as a constitutively activated mutant, interfering with sorting of membrane proteins into Golgi-derived COPI vesicles. The ARF1 (T31N) mutant instead has low affinity for GTP, acting as a dominant-negative mutant that blocks formation of COPI vesicles [7]. Regarding the SAR1 (H74L) mutation, it creates a dominant mutant variant that is insensitive to its GTPase-activating factor and thus it is fixed as a GTP-bound form. This isoform of SAR1 changes the localization of a *cis*-Golgi marker to the ER and blocks trafficking of a vacuolar protein out of the ER [3]. These mutant proteins are thought to act in a dominant fashion by titrating out factors needed for normal GTPase activity, and therefore out-competing the function of the native proteins.

To study the effect of these dominant-mutant GTPases on trafficking of CAH1 through the endomembrane system, we wished an inducible system in which expression of the mutant proteins could be controlled since constitutive expression of these mutants likely would be lethal for the plant. We induced ARF1 expression under the control of a heat-shock promoter [7] in four-weeks-old *Arabidopsis* plants. Unfortunately, ARF1 expression in leaf tissue was very low (data not shown), which in combination with the time needed for obtaining good chloroplast preparations, prompted us to seek a different experimental system. In addition, arrested protein biosynthesis by CHX indicated that turnover of the CAH1 protein was low. This could result in a concealed inhibitory effect, since CAH1 protein already located at the chloroplast would remain at nearly endogenous levels, potentially masking the effect of the mutant GTPases.

At the same time, the experimental setup had to ensure precise co-expression of CAH1 with the mutant GTPase within the cell. This requirement is a problem when using transient expression techniques, such as protoplast transfection, where only a subpopulation of the cells will take up the foreign DNA. One solution was to use fluorescently tagged mutant GTPases, and exclusively analyze cells exhibiting fluorescence. Unfortunately, previous studies on some GTPases reported that these proteins exhibits reduced activity when fluorescently tagged [15].

### Development of an Optimized 2A Co-expression System

To fulfil these criteria, we decided to optimize the 2A system in which a 16–20 amino acid long peptide used by some RNA viruses enables the synthesis of several gene products (proteins) from a single transcript, without requirement of extra-ribosomal factors [17], [19], [33]. During translation, 2A causes a premature release of the polypeptide N-terminus of 2A without stopping subsequent translation of the transcript. We reasoned that co-expression of mutant GTPases using the 2A system with epitope-tagged CAH1 would solve above mentioned obstacles. Transient expression with such constructs would ensure efficient analysis of the epitope-tagged CAH1 in a mutant GTPase background.

The 2A peptide had previously been tested in plant cells, but efficient cleavage of the 2A polyprotein was questioned, suggesting that less than 50% of the polyprotein was actually cleaved [15]. To make certain that the effect by the mutant GTPases were properly assessed, improved 2A cleavage efficiency and subsequent activity of the generated GTPases had to be demonstrated. Initially, two factors potentially influencing cleavage efficiency were tested: 1) cytosolic versus membrane-bound ribosomal translation, and 2) effect of the sequence N-terminal of 2A. The effect of these two factors had already been shown to play an important role in mammalian cells, as described by de Felipe and co-authors [34]. In a thorough study they could observe a significant discrepancy in cleavage efficiency for some 2A constructs with N-terminal exocytic signal sequences when expressed *in vitro* using cell-free translation systems compared to *in vivo* expression in HeLa cells. Interestingly, they found that the C-terminal region of EYFP, when placed N-terminal of 2A, was prone to inhibit the 2A reaction and therefore cause ‘slipstreaming’ into the ER. Upon deletion of the last 20 aa of EYFP this inhibitory effect was relieved. This made the authors conclude that for constructs with N-terminal signal peptides, the sequence immediately upstream of 2A may inhibit the 2A reaction due to interaction between the nascent protein and the translocon complex, causing ‘slipstreaming’ into the ER.

A fluorescent marker protein (CFP), transcriptionally fused to RABD2a via 2A (CFP-2A-RABD2a), constituted our starting point for developing an efficient 2A expression system (Figure 1C–F). CFP facilitated visualization *in vivo* and cleavage efficiency could be quantified by Western blotting using commercially available antibodies. Antibodies detecting RABD2a GTPase were used to verify that separation of the polypeptides was indeed specific 2A-peptide cleavage, instead of protease degradation of non-cleaved polyprotein, and that the GTPase was stable after separation from the 2A polyprotein (Figures 2 and 3).

### 2A Promotes Efficient Cleavage at the C-terminus of CFP Derivatives in Plant Cells

Interestingly, 2A-containing polyproteins tested in this work showed that intracellular targeting and the sequence N-terminal of 2A strongly affected 2A cleavage (Figure 2I and 2J). When the polypeptide N-terminal of 2A contained an ER-targeting signal sequence, cleavage efficiency was significantly increased. This effect was different from what de Felipe and colleagues could observe in mammalian cells, where addition of ER-signal sequences rather seemed to inhibit some 2A reactions [34]. Whether this effect is due to slight variation in the sequences surrounding 2A in both studies, or whether efficiency varies between mammalian and plant cells, remains to be tested. In addition, insertion of the GUS moiety between CFP and 2A resulted in almost complete cleavage although this construct is translated by cytosolic ribosomes, indicating that the sequence N-terminal of 2A is not only important when translated by membrane-bound ribosomes. Importantly, all constructs revealed expected subcellular localization of the fluorescent marker protein (Figure 2A–H and Figures S1 and S2), suggesting no significant mistargeting of the 2A-tagged markers in this study.

The reason for increased cleavage efficiency for some constructs in this study remains to be fully understood. Based on the fact that the turnover rates of the 2A constructs and the corresponding cleaved products were similar, there seemed to be no substantial difference in stability among the constructs tested (Figure S4). Although targeting information and sequence N-terminal of 2A proved to be important for cleavage efficiency in our system, functionality of the 2A peptide should be verified in each individual case.

### 2A Derived Small GTPases are Stable and Functional after 2A Cleavage

An important issue regarding the 2A technology is to determine whether the proteins resulting from cleavage of 2A containing polypeptides are stable and functional. Our results clearly showed that the levels of released RABD2a protein perfectly correlated with cleavage efficiency of the 2A constructs (Figure 2I and 2J and Figure 3), indicating that cleavage was properly carried out, released RABD2a protein was stable and low levels of fusion proteins were not due to premature termination of transcription or translation and/or degradation of the 2A C-terminal moiety, as proposed by Samalova and co-workers [15].

It has previously been shown, using approaches independent of the 2A system, that SAR1 (H74L) and RABD2a (N121I) inhibit the ER-to-Golgi anterograde transport of Golgi membrane proteins and re-locates them to the ER [3], [8], [21]. Moreover, expression of dominant mutants of the ARF1 GTPase led to disruption of the Golgi structure and its fusion to the ER, with a concomitant relocation of Golgi-resident proteins to the ER. Co-expression of ARF1 (T31N) led to re-absorbance of markers of *cis*-, *medial*-, and *trans*-Golgi into the ER [23], [35]. However, co-expression of ARF1 (Q71L) had a much weaker effect on the distribution of Golgi markers [35].

In our study, we were able to reproduce similar effects on ER-to-Golgi transport using 2A derived dominant mutant isoforms (Figures 4 and 5). Co-expression of wild type RABD2a and SAR1 with secreted SP-CFP-2A and Golgi targeted GS-CFP-2A, did not alter the distribution of these marker proteins. On the contrary, the respective dominant mutant protein caused blockage of the ER-to-Golgi anterograde transport, as evidenced by inhibited secretion of SP-CFP-2A and blockage of GS-YFP-2A or GS-CFP-2A in the ER (Figures 4 and 5A–D). Additionally, and in agreement with observations by other groups, the GTP- and GDP-locked ARF1 mutants originating from our 2A constructs showed a differential effect on the Golgi marker GS-CFP-2A. The ARF1 (T31N) mutant (GDP-locked) caused a relocation of GS-CFP-2A to the ER in protoplasts. In addition, a subpopulation of the transfected protoplasts showed fluorescent vacuole-like structures potentially indicating mistargeting of the Golgi marker when ER to Golgi trafficking was blocked (Figure 5E–H and Figure S7). On the contrary, ARF1 (Q71L) (GTP-locked) caused a weaker relocation of the GS-CFP-2A marker to the ER, but instead an altered Golgi appearance, similar to previous reports (Figure 5I–K) [4]. In conclusion, our results clearly showed that the dominant-mutant forms of the three small GTPases tested in this work exerted the expected interference with membrane trafficking after 2A cleavage.

### Processing of 2A Peptide Fused ORFs

Our study revealed that the CFP-containing product N-terminal of 2A, or the non-cleaved polyprotein, was partly processed to a lower molecular mass variant. This modified isoform was consistently migrating at about 28 kDa upon SDS polyacrylamide gel separation. Peptide extensions N-terminal of the CFP protein, or between CFP and 2A, did not alter the size of the processed form (Figure S5 and Figure 2I), indicating processing at both the N- and C-terminus of CFP. This effect has been previously observed when Tamura and co-workers were addressing difficulties to observe GFP in vacuoles [30]. Vacuolar targeted GFP with a theoretical size of 30 kDa was proteolytically processed to a 27-kDa form upon entering the vacuole. Both N- and C-terminally tagged GFP was processed, indicating that GFP modification in the vacuole takes place at both termini. The 27 kDa variant reported by Tamura *et al*. strongly resembled our 28 kDa band. However, no clear CFP fluorescence could be detected in the vacuoles of transfected protoplasts. In a previous report by Samalova and co-workers [15] it was postulated that the 2A moiety itself could act as a vacuolar targeting signal leading to mislocalization of 2A containing proteins. This fact could preclude the use of 2A. Importantly, our data showed that the detected 28 kDa degradation product was not unique to CFP tagged with 2A but was also observed in CFP constructs lacking 2A. Moreover, our analysis showed that the 28 kDa product represented only a small portion of the total pool of CFP containing polypeptides, suggesting that this does not significantly interfere with application of the 2A technology.

### RABD2a, SAR1, and ARF1 Proteins are Involved in the Trafficking of CAH1 through the Endomembrane System

Efficient cleavage of 2A constructs, together with high stability of the released proteins, and correct dominant-behaviour after cleavage prompted us to use the 2A technology to test small GTPases dominant mutant derivatives on the plant intracellular trafficking routes. In an initial attempt to characterize trafficking of CAH1 through the endomembrane system, we tested if the optimized 2A system could be used to interfere with ER to Golgi transport.

HA-tagged CAH1 was fused in frame to wild type and dominant mutant versions of the GTPases RABD2a, SAR1, and ARF1 separated by 2A. Although the highest cleavage efficiency was achieved when GUS was located N-terminal of 2A (Figure 2), cleavage was also efficient when translation was performed by ER-bound ribosomes. Since HACAH1 already possessed an N-terminal ER signal sequence, we speculated that cleavage efficiency would be sufficient without GUS present. Indeed, the resulting HACAH1-2A-RABD2a/SAR1/ARF1 constructs showed high cleavage, confirming efficient function of 2A when translated by ER-bound ribosomes. Cleavage was efficient in transfected protoplasts as well as when expressed *in planta*, demonstrating the versatility of the 2A system (Figure 6A–E).

Although different glycosylated isoforms of the HACAH1-2A protein could be found, some of them were resistant to Endo H as previously shown for the native CAH1 protein in *Arabidopsis* chloroplasts [9], [24]. We therefore used resistance or susceptibility to Endo H as a reported marker to study trafficking of the CAH1 protein [8]. Using this approach we showed a pronounced effect of the dominant mutants of both RABD2a and SAR1 proteins on the trafficking of CAH1 through the endomembrane system (Figure 6A, 6B and 6F). In agreement with previous studies on different proteins [3], [6], [8], we demonstrated that these two dominant inhibitory mutants blocked the ER-to-Golgi anterograde transport of CAH1, as observed from accumulation of the Endo H sensitive, presumably ER-located isoform. Expression of ARF1 (T31N) mutant, but not ARF1 (Q71L), increased susceptibility of the CAH1 protein to Endo H (Figure 6C and 6F), indicating that inhibition of the retrograde transport pathway by ARF1 (T31N) may block transport of CAH1 in the ER, preventing further N-glycan processing and rendering a protein more susceptible to Endo H. It is also plausible that other proteins, e.g. Golgi N-glycan processing enzymes, would also relocate to the ER and could perform their action there, converting the high-mannose type N-glycans of ER-resident proteins into Endo H resistant forms [36]. The effect of ARF1 mutants on CAH1 trafficking would then be difficult to evaluate using our Endo H assay. However, our results are consistent with data published by Kitajima and co-workers [13], showing that trafficking of a plastid targeted Amyl-1 GFP fusion in onion cells was more affected by ARF1 (T31N) than ARF1 (Q71L). In addition, stronger effect of the SAR1 (H74L) mutant version than that of the ARF1 (T31N) mutant was also reported. We could also show that the effect of the mutant RABD2a (N121I) was similar to that of SAR1 (H74L), suggesting that the effect on phenotype is more severe when the mutant acts at an early step of the trafficking pathway.

To confirm that the 2A system was also functional in an *in planta* model system, HACAH1-2A-RABD2a/SAR1/ARF1 were expressed in *Nicotiana benthamiana* leaves using *Agrobacterium*-mediated infiltration. Not only did we observe cleavage efficiencies very similar to those measured in transiently transfected protoplasts, the effect of the mutated GTPases could also be reproduced *in planta*, although the phenotype was slightly reduced (Figure 6D, 6E and 6G). This demonstrates the potential of the 2A system as a tool for protein trafficking studies.

One pitfall with transient expression techniques, such as protoplast transfection, is that analysis of a heterogeneous population of transfected and non-transfected cells often masks the mutant phenotype. If the effect one aims to study is weak, or the transfection efficiency is low, the resulting effect might be hidden by the non-transfected cells. Under such circumstances, it is desirable to sort out transfected cells to obtain a homogeneous mutant population. The 2A system also offers a possible solution to such problems, since protoplasts transfected with the Golgi labelling GS-CFP-2A-RABD2a construct could be analyzed by flow cytometry, and satisfactorily separated in two subpopulations (transfected and non-transfected cells). Examination by fluorescence microscopy (data not shown) and immunoblot analysis of the corresponding protein extracts (Figure S9), revealed successful sorting. Therefore, transient protoplast transfection using the 2A system combined with fluorescence-activated cell sorting (FACS) can give a homogeneous mutant population of plant protoplasts in less than 24 h.

In conclusion, this work shows how an optimized application of the 2A system can be used for fast, versatile and simultaneous co-expression of individual proteins in plant protoplasts and cells. Low activity normally seen by tagged proteins, due to the intrinsic properties of the protein of interest, can be avoided by the ability of 2A to generate individual proteins from a single transcript. This property can be exploited in a multitude of methods, here exemplified by biochemical assays, live-cell imaging and cell sorting.

Based on data obtained using the improved 2A mediated co-expression system, we conclude that early trafficking of the plastid N-glycoprotein CAH1 depends on canonical vesicular transport mechanisms. Identification of the specific targeting domains within the CAH1 protein, and the factors responsible for targeting and transport of the protein from the Golgi to the chloroplast will provide further insight into the mechanism underlying the targeting of N-glycoproteins to the chloroplast.

## Materials and Methods

### Molecular Cloning and Generation of 2A Containing Constructs

DNA coding sequences were constructed using standard cloning techniques and polymerase chain reaction (PCR) to generate the in-frame protein fusion junctions. The coding sequence for enhanced cyan fluorescent protein (CFP) from pAVA574 [37] was excised with *BglII* and *NcoI* and was inserted to the corresponding site of pPE1000 [38] creating pSB-CFP containing CFP under the control of the double Cauliflower mosaic virus 35S promoter of pPE1000. A double-stranded oligonucleotide coding for 2A was constructed by PCR using the primers 2Afor/*BglII* (AAAAAAGATCTCAGCTGTTGAATTTTGACCTTCTTAAGCTTGCGGGAGACG) and 2Arev/*SalI* (AAAAAGTCGACGGGGGCCCAGGGTTGGACTCGACGTCTCCCGCAAGCTTAA). The corresponding PCR fragment was purified by phenol/chloroform extraction, precipitated using ethanol, digested and subcloned as a *BglII-SalI* fragment into the corresponding site of pSB-CFP creating pSB-CFP-2A.

pSB-CFP-2A-RABD2a/RABD2a (N121I) were created by PCR amplification of the RABD2a/RABD2a (N121I) coding sequences from pVKHEn6-Ara5mΔGUS [8] using D2afor/*SalI* (AAAGTCGACCATGAATCCTGAGTACGACT) and D2arev/*BamHI* (AAAAAGGATCCTCAAGTTGAGCAGCAGCCG). The PCR product was digested with *SalI* and *BamHI* and was inserted into the corresponding site of pSB-CFP-2A to create pSB-CFP-2A-RABD2a/RABD2a (N121I). The coding sequences for wild type and GTP-locked SAR1 were PCR amplified from SAR1-YFP and SAR1-GTP-YFP respectively [28] using SAR1for/*SalI* (AAAAGTCGACAATGTTCTTGGTAGATTGGT) and SAR1rev/*BamHI* (TTGGATCCTTACTTGATATACTGAGACATC). The PCR product was inserted into *SalI* and *BamHI* digested pSB-CFP-2A-RABD2a to create pSB-CFP-2A-SAR1/SAR1 (H74L). To create pSB-CFP-2A-ARF1 containing wt, GDP- and GTP-locked ARF1, the coding sequence for ARF1 was PCR amplified from 35S-ARF1-WT-EGFP, 35S-ARF1-T31N-EGFP and 35S-ARF1-Q71L-EGFP [7] respectively using ARF1for/*SalI* (AAAGTCGACAATGGGGTTGTCATTCGGAAA) and ARF1rev/*BamHI* (TTGGATCCCTATGCCTTGCTTGCGATGTTG). The PCR product was inserted into *SalI* and *BamHI* digested pSB-CFP-2A-RABD2a to create pSB-CFP-2A-ARF1/ARF1 (T31N)/ARF1 (Q71L).

The DNA sequences coding for the transmembrane-stem regions of *Arabidopsis thaliana N*-acetylglucosaminyl transferase I, fused to enhanced cyan/yellow fluorescent protein (GS-CFP/GS-YFP) under control of the enhanced 35S promoter, were excised from respective pGreenII vector [20] using *XhoI* and *BsrGI*. The corresponding DNA fragment was used to replace the *XhoI-BsrGI* fragment of pSB-CFP-2A-RABD2a/RABD2a (N121I), pSB-CFP-2A-SAR1/SAR (H74L) and pSB-CFP-2A-ARF1/ARF1 (T31N)/ARF1 (Q71L) to create pSB-GS-CFP-2A-RABD2a/RABD2a (N121I) and pSB-GS-YFP-2A-RABD2a/RABD2a (N121I), pSB-GS-CFP-2A-SAR1/SAR (H74L) and pSB-GS-CFP-2A-ARF1/ARF1 (T31N)/ARF1 (Q71L) respectively.

The CFP-2A-RABD2a/RABD2a (N121I) coding region from pSB-CFP-2A-RABD2a/RABD2a (N121I) was excised with *NcoI* and *NotI* and sub-cloned into the corresponding site of CaMV35S-(1-40)CAH1-sGFP(S65T) [9] creating pSB-SP-CFP-2A-RABD2a/RABD2a (N121I), where the 40 amino acid N-terminal signal peptide containing sequence from CAH1 is fused in-frame to CFP-2A-RABD2a/RABD2a (N121I) under the control of the 35S promoter.

To create pSB-CFPGUS-2A-RABD2a, the coding region of β-glucuronidase (GUS) was amplified by PCR from pKGWFS7 [39] using the primers GUSfor/*BglII* (ACAAGAGATCTATGTTACGTCCTGTAGAAA) and GUSrev/*BglII* (CGCCAAGATCTTTGTTTGCCTCCCTGCTGC). The PCR product was digested using *BglII* and inserted into *BglII* digested pSB-CFP-2A-RABD2a, to create pSB-CFPGUS-2A-RABD2a.

The N-terminally HA tagged CAH1 sequence [24] was PCR amplified with ForCAH1/*BspHI* (AAAATCATGAATGAAGATTATGATGATGA) and RevCAH1/*BsrGI* (TTTTGTACAGATTGGGTTTTTTCTTTTTGT), digested by *BspHI* and *BsrGI*, and sub-cloned into *NcoI* and *BsrGI* digested pSB-CFP-2A-RABD2a/RABD2a (N121I) to create pSB-HACAH1-2A-RABD2a/RABD2a (N121I). The double 35S promoter HACAH1 sequence of pSB-HACAH1-2A-RABD2a was excised using *XhoI* and *BsrGI* and used to replace the double 35S promoter CFP sequence of pSB-CFP-2A-SAR1/SAR1 (H74L) and pSB-CFP-2A-ARF1/ARF1 (T31N)/ARF1 (Q71L), to create pSB-HACAH1-2A-SAR1/SAR1 (H74L) and pSB-HACAH1-2A-ARF1/ARF1 (T31N)/ARF1 (Q71L).

Finally, CFP-2A-RABD2a was excised from pSB-GS-CFP-2A-RABD2a with *NcoI* and *BamHI*, and replaced with a PCR amplified CFP-2A-Stop fragment from pSB-CFP-2A-RABD2a using For-35S (GCAAGACCCTTCCTCTATA) and 2Arevstop/*BamHI* (CATGGATCCTCAGGGCCCAGGGTTGGACTC), digested with the same restriction enzymes to create pSB-GS-CFP-2A.

Plasmid DNA from the different *Escherichia coli* clones was amplified by PCR using the following primers: pSB pSB-HACAH1-2A-RABD2a/RABD2a (N121I) with ForKpnI/SP-HC (AAGGTACCATGAAGATTATGATGATGATTA) and RevSacI/RabD2a (GAACGGCTGCTGCTCAACTTGAGAGCTCAA); pSB-HACAH1-2A-SAR1/SAR1 (H74L) with ForKpnI/SP-HC and RevSacI/Sar1 (GATGTCTCAGTATATCAAGTAAGAGCTCAA); and pSB-HACAH1-2A-ARF1/ARF1 (T31N)/ARF1 (Q71L) with ForKpnI/SP-HC and RevSacI/Arf1 (CAACATCGCAAGCAAGGCATAGGAGCTCAA).

The corresponding PCR fragment was purified with QIAquick PCR Purification Kit (QIAGEN, Germany), digested and subcloned as a KpnI-SacI fragment into the corresponding site of pMDC32-GFP [40], creating pMDC-HACAH1-2A-RABD2a/RABD2a (N121I), pMDC-HACAH1-2A-SAR1/SAR1 (H74L) and pMDC-HACAH1–2A-ARF1/ARF1 (T31N)/ARF1 (Q71L) respectively. These binary vectors were electrotransformed at 2.5 kV with GenePulser (BIO-RAD, USA) in *Agrobacterium tumefaciens* strain GV3101 (pM90) and grown in YEB plates supplemented with the appropriate antibiotics for 48h at 28°C. Positive colonies were selected and checked by PCR.

The H2B-Cherry nuclear marker was created by amplifying the *Arabidopsis* histone H2B (At5g22880) cDNA and cloned as a SalI-KpnI fragment in translational fusion with the mCherry protein in the pmCherry vector (Clontech, USA). The H2B-mCherry was then moved as a SalI-XbaI fragment into the plant expression vector pRT100 [41].

The DNA sequences of all constructions were verified by DNA sequence analysis and/or restriction analysis to ensure correct sequence and reading frame.

### Establishment of Stably Transformed Arabidopsis Cell Suspension Cultures and Transient Expression in Arabidopsis Thaliana Protoplasts

*Arabidopsis thaliana* cell suspension culture (ecotype Columbia-0), was grown as described [24]. *Arabidopsis* cells were maintained by weekly sub-culturing of 8 ml culture into 42 ml fresh medium in 250 ml Erlenmeyer flasks rotated at 120 rpm at 16 h light (22°C) and 8 h dark (18°C) cycle. *Agrobacterium* mediated plant cell culture transformation, as well as transient expression experiments in protoplasts, was performed as previously described [24]. 5×10^5^ protoplasts were transfected with 5 to 7 µg plasmid DNA.

### Transient Expression in Nicotiana Benthamiana Plants

*Nicotiana benthamiana* plants, were transformed with the different constructs using *A. tumefaciens* inoculation, according to Sparkes *et al*., [42] and grown in at 16 h light (22°C) and 8 h dark (18°C) cycle. Transient co-expression of p19 plasmid was used to avoid gene silencing [43]. The agroinfiltrated leaves were harvested at 2 dpi and protein extracts were done as described below.

### Preparation of Protein Extracts and Blot Analysis

Total protein extract was prepared from protoplasts 24 h post-transformation. Protoplasts were pelleted by centrifugation first at 190 g for 7 min to remove most of the protoplast medium, followed by a 30 sec centrifugation at 2000 g in a microcentrifuge to completely remove residual medium. Protoplasts prepared for SDS-PAGE [44] by resuspending protoplasts in 2× sample buffer (125 mM Tris-HCl, 4% (w/v) SDS, 20% (v/v) glycerol, 0.006% (w/v) bromophenol blue, 0.3 M DTT, pH 6.8) and heated at 95°C for 5 min.

Proteins secreted from transfected protoplasts were precipitated using trichloroacetic acid (TCA) and sodium-deoxycholate (DOC). Forty-five µl 0,15% (w/v) DOC was added to 300 µl protoplast media centrifuged at maximum speed in a microcentrifuge for 30 min at 4°C, vortexed and incubated for 10 min at room temperature (RT). Proteins were precipitated by adding 45 ul 72% (w/v) TCA, vortexed and incubated for 2 h on ice. Proteins were pelleted by centrifugation for 15 min at 4°C and washed with 800 ul acetone (−20°C), air dried and resuspended in sample buffer.

For Endo H treatment, one agroinfiltrated leaf of *Nicotiana benthamiana* was grinded in 1 ml extraction buffer (25 mM Tris-HCl pH 7.8, 10 mM MgCl_2_, 5 mM EGTA, 2 mM DTT, 10% (v/v) glycerol, 75 mM NaCl, 0.2% (v/v) IgePal-630, 1 mM benzamidine, and protease inhibitor cocktail (PIC, Sigma).

Proteins were separated by SDS-PAGE, followed by immunoblot analysis as previously described [24]. Proteins were probed with α-GFP antibodies (GFP (B-2), sc-9996, Santa Cruz Biotechnology, Inc. CA 95060, USA), α-HA antibodies (HA11, Nordic Biosite AB, Täby, Sweden), α-BiP (Hsc70 from Nordic Biosite AB, Sweden) or with antibodies detecting RABD2 kindly provided by Dr. Ian Moore (Oxford, UK). All primary antibodies were used at 1∶1000 dilutions and incubated overnight at 4°C in TBS/Tween-20 supplemented with 2% (w/v) non-fat dry milk.

### Deglycosylation Using Endo H

Protoplasts were pelleted and resuspended in 30 ul extraction buffer and vortexed for 30 sec. Protoplasts were frozen in liquid N_2_, and thawed on ice. Deglycosylation of both protoplast and *N. benthamiana* leaf extracts was performed using Endo H enzyme (New England Biolabs, Hertfordshire, UK). Briefly, 1 ul denaturing buffer was added to 9 ul soluble extract after centrifugation in a microcentrifuge and heated at 100°C for 10 min. The reaction volume was brought to 20 ul by addition of 500 U Endo H, G5 reaction buffer and PIC. Deglycosylation was performed over night at 37°C. Reaction was stopped and samples prepared for SDS-PAGE by addition of 20 ul 2× sample buffer and heated 5 min at 95°C.

### Protein Quantification and Cleavage Efficiency Analysis

Proteins were separated using SDS-PAGE, blotted and quantified as previously described [24]. Cleavage analysis was estimated from a minimum of four individual transfections. Duplicates of different volumes of the protein sample were separated using SDS-PAGE, followed by detection using GFP antibodies. Cleavage efficiency was calculated from the ratio of the sample volumes needed to achieve an equal immunoreaction for cleaved and non-cleaved polypeptide.

### Confocal Analysis

Fluorescent proteins were visualised using a Leica TCS-SP2 laser scanning confocal microscope (Leica Microsystems, Heidelberg, Germany) with a 63×1.4 NA oil-objective lens at RT and photographed using Leica confocal software v.2.61 (Leica) about 24 h post-transformation [9]. ER-Cherry [45], H2B-Cherry and GmMan-tdT [46] were excited with a 561 nm laser and fluorescence detected between 570–630 nm. GFP was excited with a 488 nm argon laser and fluorescence detected between 505–550 nm. CFP was excited with a 405 nm diode laser and fluorescence detected between 465–510 nm. YFP was excited with a 514 nm argon laser and fluorescence detected between 520–550 nm. CFP and YFP in co-transfected cells was verified by lambda scans and recorded in sequential scan mode to eliminate eventual fluorescence overlap.

The Pearson's coefficient was used to estimate the level of co-localization of SP-CFP-2A and GS-YFP-2A. Images were captured from 20 protoplasts each and the Pearson's coefficient was calculated using the JACoP plug-in [29] of the ImageJ software [47].

Golgi size was estimated from protoplasts transfected with GS-CFP-2A-ARF1/ARF1 (Q71L) using the “Analyze Particles” tool in ImageJ [47]. Only Golgi labelling above a certain intensity and size threshold was analyzed in order to remove ER-like and non-specific background fluorescence. Pixel area was converted to physical area using the information from the Leica output file.

## Supporting Information

Figure S1 **2A constructs correctly co-localized with known markers.** Confocal (a and b) and bright field (d) images of *Arabidopsis* protoplasts transiently co-expressing different markers. Merged image (c) shows co-localization (white) of constructs shown in (a) and (b). (A) Cytosolic CFP-2A-RABD2a (a) and GFP (b). (B) Cytosolic CFP-2A-RABD2a (a) and nuclear H2B-Cherry (b). (C) Cytosolic CFP-2A-RABD2a (a) and ER-localized ER-Cherry (b). (D) Cytosolic CFPGUS-2A-RABD2a (a) and GFP (b). (E) Cytosolic CFPGUS-2A-RABD2a (a) and nuclear H2B-Cherry (b). (F) Cytosolic CFPGUS-2A-RABD2a (a) and ER-localized ER-Cherry (b). (G) ER-localized SP-CFP-2A-RABD2a (a) and cytosolic GFP (b). (H) ER-localized SP-CFP-2A-RABD2a (a) and ER-Cherry (b). (I) ER-localized SP-CFP-2A-RABD2a (a) and Golgi-localized GmManI-tdT (b). (J) Golgi-localized GS-CFP-2A-RABD2a (a) and GmManI-tdT (b). Bars = 10 µm.
(TIF)

Figure S2 **Presence of 2A does not affect the final targeting of GS-YFP-2A.** Confocal (A and B) and bright field (D) images of *Arabidopsis* protoplasts transiently co-expressing GS-CFP (A) and GS-YFP-2A-RABD2a (B). Merged image (C) shows co-localization (white) of both constructs in Golgi-like structures. Bars = 5 µm.
(TIF)

Figure S3 **The cleavage efficiency of CFPGUS-2A-RABD2a is high.** Detailed immunoblot analysis of protein extracts from *Arabidopsis* protoplasts transiently transfected with water (−) or CFPGUS-2A-RABD2a, using anti-RABD (A) or anti-GFP (B) antiserum. Cleaved CFPGUS-2A (*) and non-cleaved 2A polyprotein CFPGUS-2A-RABD2a (**) are indicated.
(TIF)

Figure S4 **Cleavage efficiency is not affected by protein turnover.** No significant difference in cleavage efficiency could be seen upon cycloheximide (CHX) treatment in intracellular protein extracts, indicating that the estimated cleavage efficiency (Figure 2J) is not due to degradation of non-cleaved polypeptide. (A) CFP-2A-RABD2a (wt), (B) SP-CFP-2A-RABD2a (wt), (C) GS-CFP-2A-RABD2a (wt) and (D) CFPGUS-2A-RABD2a (wt). Numbers indicated hours after CHX addition (0, 2, 4 and 6 h). Cleaved polypeptide (*), non-cleaved 2A polyprotein (**) and the putative degradation product (−) are indicated.
(TIF)

Figure S5 **CFP degradation product is not unique to 2A constructs.** (A and B) Scheme over GS-CFP (A) and GS-CFP-2A (B). (C) Anti-GFP immunoblot analysis of protein extracts from *Arabidopsis* protoplasts transiently expressing GS-CFP, GS-CFP-2A, and GS-CFP-2A-RABD2a (shown in Figure 1E). The 28 kDa degradation product (−) is detected in the different protein extracts, regardless of the construct being expressed.
(TIF)

Figure S6 **GS-CFP-2A degradation product is affected by BFA and RABD2a (N121I).** (A) Anti-GFP immunoblot analysis of protein extracts from *Arabidopsis* protoplasts transiently expressing GS-CFP-2A-RABD2a (wt) or GS-CFP-2A-RABD2a (N121I). BFA was added to protoplasts expressing GS-CFP-2A-RABD2a (wt). Non-cleaved full length GS-CFP-2A-RABD2a (**), cleaved GS-CFP-2A (*) and putative degradation product (−) are indicated. (B) Quantification of the intensity of the 28 kDa band. The ratio of the 28 kDa degradation product (−) and 2A released GS-CFP-2A (*) was calculated. Error bars show standard error (n = 4, *** = p<0.001).
(TIF)

Figure S7 **Accumulation of GS-CFP-2A in vacuole like structures when co-expressed with ARF1 (T31N).** Bright field (A) and confocal (B) images of *Arabidopsis* protoplast transiently expressing GS-CFP-2A-ARF1 (T31N). In addition to the phenotype shown in Figure 5G–H, some protoplasts showed CFP fluorescence in vacuole like structures as shown here. Bars = 5 µm.
(TIF)

Figure S8 **Endo H is a valid marker for protein trafficking.** (A) Immunoblot analysis of ultracentrifuged protein extracts from *Nicotiana benthamiana* transiently expressing HACAH1-2A-RABD2a (wt) using anti-HA, anti-Rubisco and anti-BiP antibodies. Soluble stroma-containing and pelleted ER-containing microsome fractions are indicated. Samples were subjected (+) or not (−) to Endo H treatment. Non-cleaved full-length (**) and cleaved (*) products, as well as resistant (black triangle) and sensitive (white triangle) bands are indicated.
(TIF)

Figure S9 **Sorting of 2A polyprotein transfected protoplasts using FACS.** (A) Scheme of fluorescent-activated cell sorting (FACS) of protoplasts transfected with 2A polyprotein in order to obtain a homogenous mutant population. Protoplasts were transfected with GS-CFP-2A-RABD2a vector (v), resulting in a heterogeneous mixture of transfected (v, about 10% of total population, data not shown) or non-transfected cells (−). Protoplasts were sorted into two populations based on the level of CFP fluorescence (v or -, respectively). (B) Immunoblot analysis of total protoplast extracts from sorted cells transfected with GS-CFP-2A-RABD2a (v/−, transfected/non-transfected respectively) using anti-BiP (loading control) or anti-GFP antiserum. Non-cleaved GS-CFP-2A-RABD2a (**) and released GS-CFP-2A (*) are indicated.
(TIF)

Table S1 **Intracellular distribution of HACAH1 in Col-0** ***Arabidopsis thaliana*** **suspension cultured cells.** Immunogold labeling density over several sub-cellular compartments was estimated in both wild type and HACAH1 stably transformed Col-0 *Arabidopsis thaliana* suspension cells using HA antiserum.
(DOCX)

Text S1 **Supporting Material and Methods.**
(DOCX)
