# Supplementary figures and images for: Use of the Foot-and-Mouth Disease Virus 2A Peptide Co-Expression System to Study Intracellular Protein Trafficking in Arabidopsis
Source: PLoS One. 2012 Dec 14;7(12):e51973. doi: 10.1371/journal.pone.0051973 (PMC3522588; doi:10.1371/journal.pone.0051973)

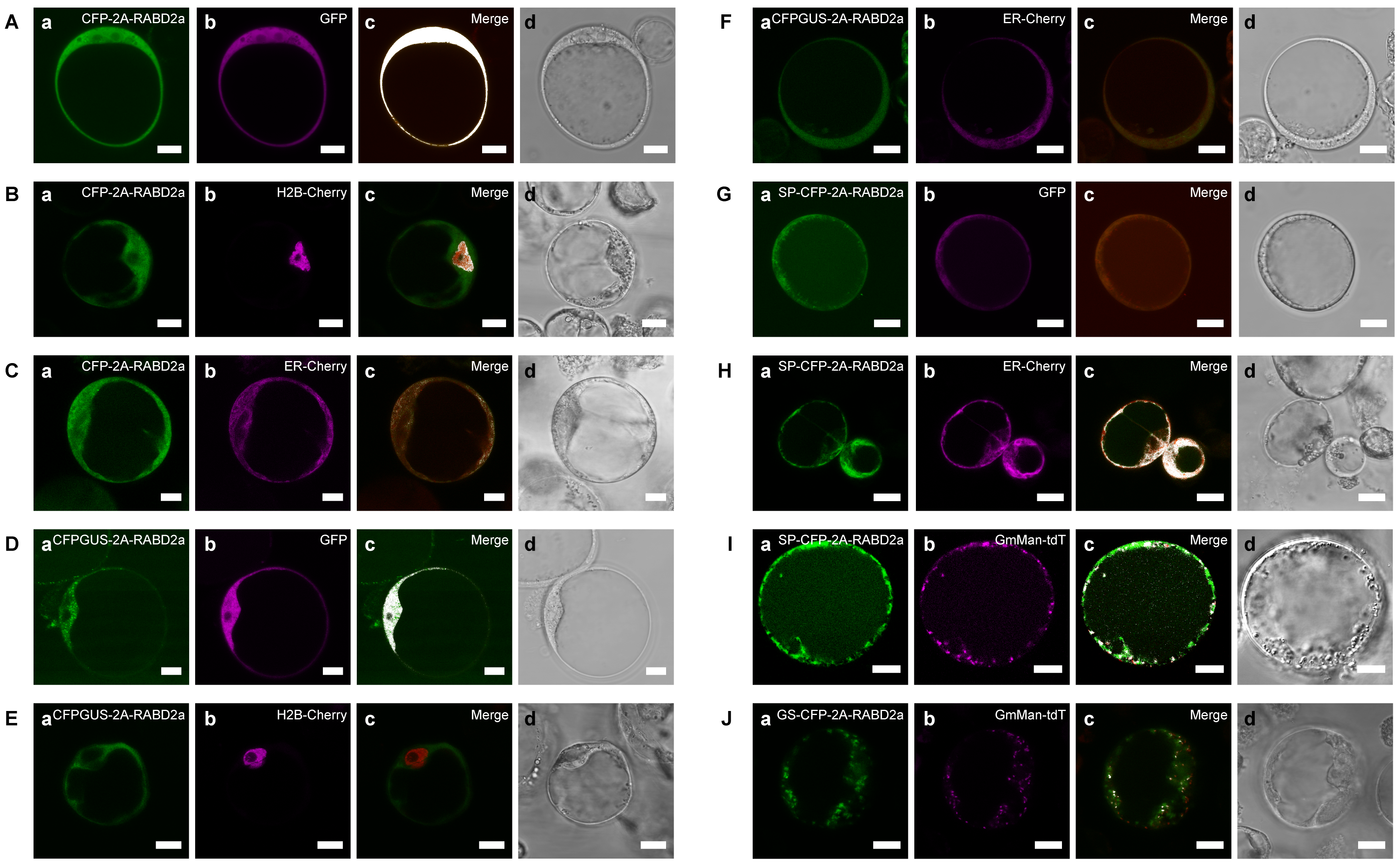

Supplement: Figure S1 — 2A constructs correctly co-localized with known markers. Confocal (a and b) and bright field (d) images of Arabidopsis protoplasts transiently co-expressing different markers. Merged image (c) shows co-localization (white) of constructs shown in (a) and (b). (A) Cytosolic CFP-2A-RABD2a (a) and GFP (b). (B) Cytosolic CFP-2A-RABD2a (a) and nuclear H2B-Cherry (b). (C) Cytosolic CFP-2A-RABD2a (a) and ER-localized ER-Cherry (b). (D) Cytosolic CFPGUS-2A-RABD2a (a) and GFP (b). (E) Cytosolic CFPGUS-2A-RABD2a (a) and nuclear H2B-Cherry (b). (F) Cytosolic CFPGUS-2A-RABD2a (a) and ER-localized ER-Cherry (b). (G) ER-localized SP-CFP-2A-RABD2a (a) and cytosolic GFP (b). (H) ER-localized SP-CFP-2A-RABD2a (a) and ER-Cherry (b). (I) ER-localized SP-CFP-2A-RABD2a (a) and Golgi-localized GmManI-tdT (b). (J) Golgi-localized GS-CFP-2A-RABD2a (a) and GmManI-tdT (b). Bars = 10 µm. (TIF) [file pone.0051973.s001.tif]

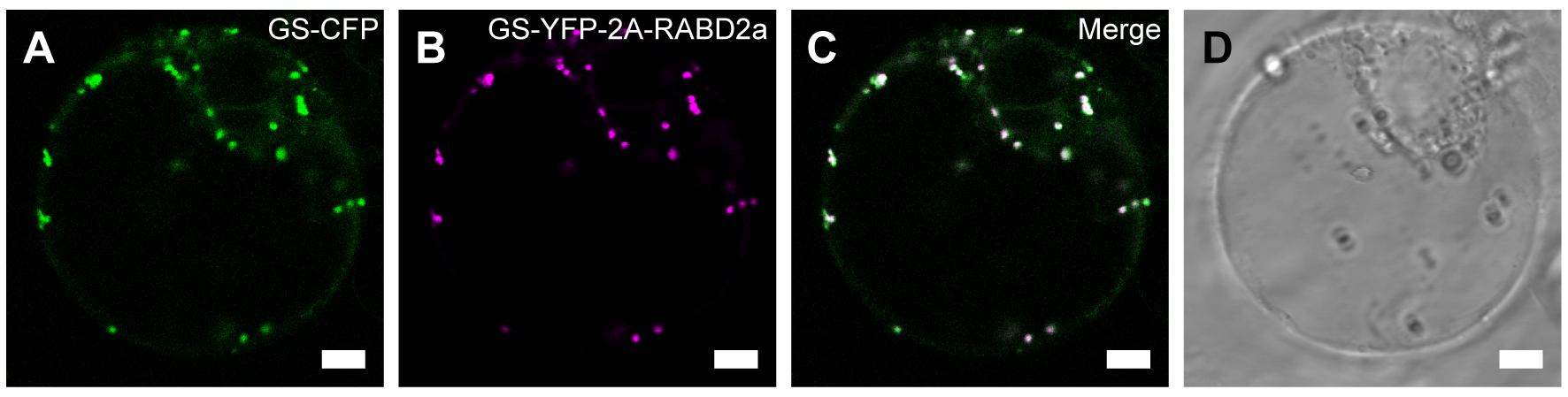

Supplement: Figure S2 — Presence of 2A does not affect the final targeting of GS-YFP-2A. Confocal (A and B) and bright field (D) images of Arabidopsis protoplasts transiently co-expressing GS-CFP (A) and GS-YFP-2A-RABD2a (B). Merged image (C) shows co-localization (white) of both constructs in Golgi-like structures. Bars = 5 µm. (TIF) [file pone.0051973.s002.tif]

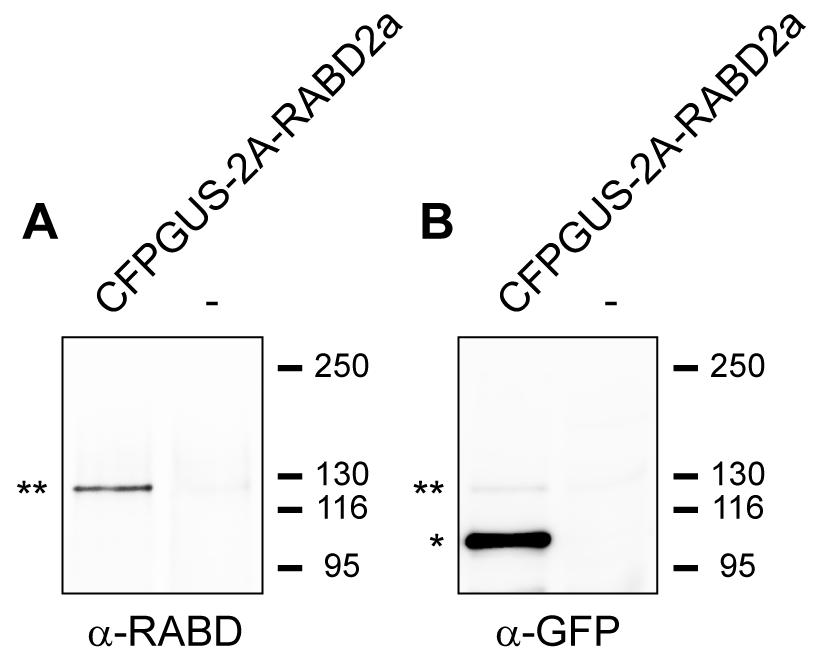

Supplement: Figure S3 — The cleavage efficiency of CFPGUS-2A-RABD2a is high. Detailed immunoblot analysis of protein extracts from Arabidopsis protoplasts transiently transfected with water (−) or CFPGUS-2A-RABD2a, using anti-RABD (A) or anti-GFP (B) antiserum. Cleaved CFPGUS-2A (*) and non-cleaved 2A polyprotein CFPGUS-2A-RABD2a (**) are indicated. (TIF) [file pone.0051973.s003.tif]

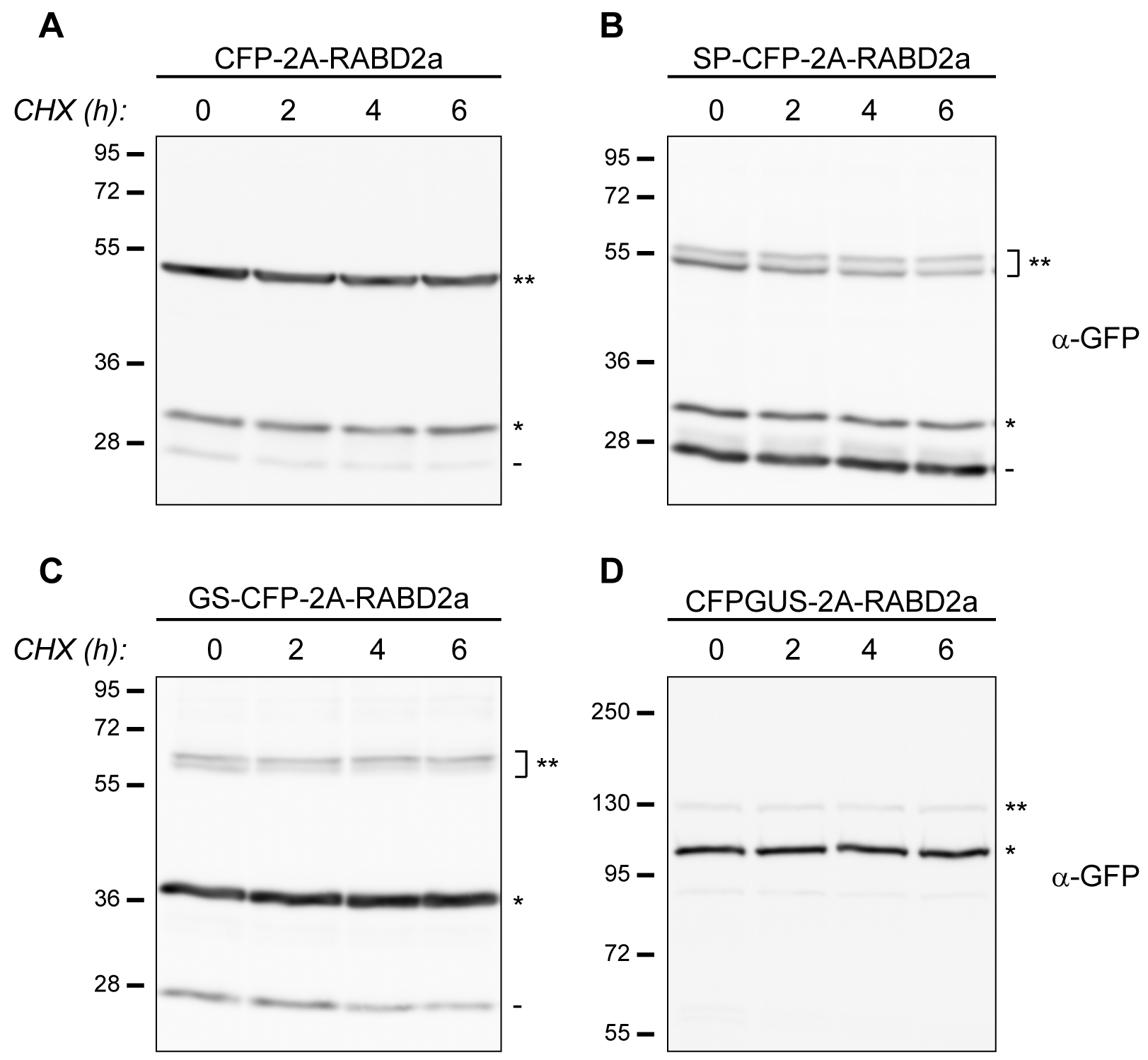

Supplement: Figure S4 — Cleavage efficiency is not affected by protein turnover. No significant difference in cleavage efficiency could be seen upon cycloheximide (CHX) treatment in intracellular protein extracts, indicating that the estimated cleavage efficiency (Figure 2J) is not due to degradation of non-cleaved polypeptide. (A) CFP-2A-RABD2a (wt), (B) SP-CFP-2A-RABD2a (wt), (C) GS-CFP-2A-RABD2a (wt) and (D) CFPGUS-2A-RABD2a (wt). Numbers indicated hours after CHX addition (0, 2, 4 and 6 h). Cleaved polypeptide (*), non-cleaved 2A polyprotein (**) and the putative degradation product (−) are indicated. (TIF) [file pone.0051973.s004.tif]

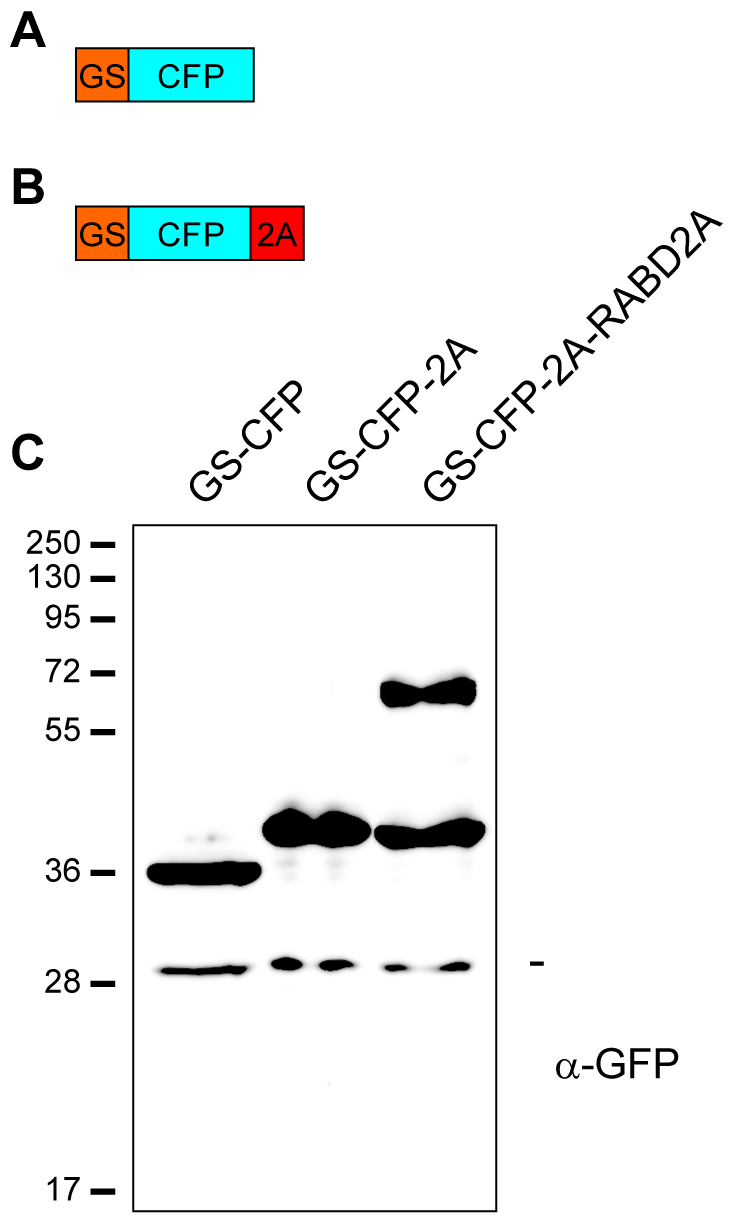

Supplement: Figure S5 — CFP degradation product is not unique to 2A constructs. (A and B) Scheme over GS-CFP (A) and GS-CFP-2A (B). (C) Anti-GFP immunoblot analysis of protein extracts from Arabidopsis protoplasts transiently expressing GS-CFP, GS-CFP-2A, and GS-CFP-2A-RABD2a (shown in Figure 1E). The 28 kDa degradation product (−) is detected in the different protein extracts, regardless of the construct being expressed. (TIF) [file pone.0051973.s005.tif]

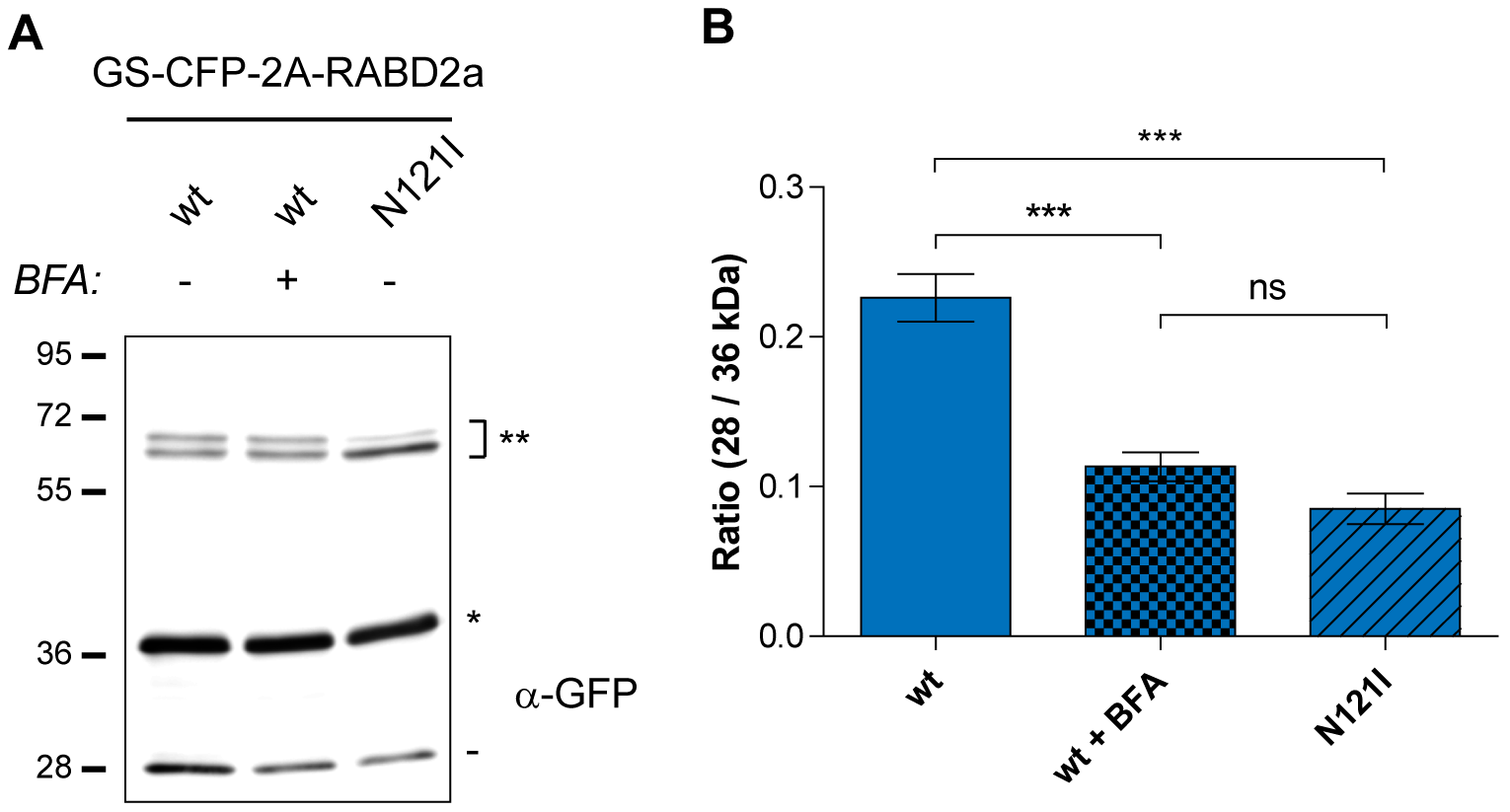

Supplement: Figure S6 — GS-CFP-2A degradation product is affected by BFA and RABD2a (N121I). (A) Anti-GFP immunoblot analysis of protein extracts from Arabidopsis protoplasts transiently expressing GS-CFP-2A-RABD2a (wt) or GS-CFP-2A-RABD2a (N121I). BFA was added to protoplasts expressing GS-CFP-2A-RABD2a (wt). Non-cleaved full length GS-CFP-2A-RABD2a (**), cleaved GS-CFP-2A (*) and putative degradation product (−) are indicated. (B) Quantification of the intensity of the 28 kDa band. The ratio of the 28 kDa degradation product (−) and 2A released GS-CFP-2A (*) was calculated. Error bars show standard error (n = 4, *** = p<0.001). (TIF) [file pone.0051973.s006.tif]

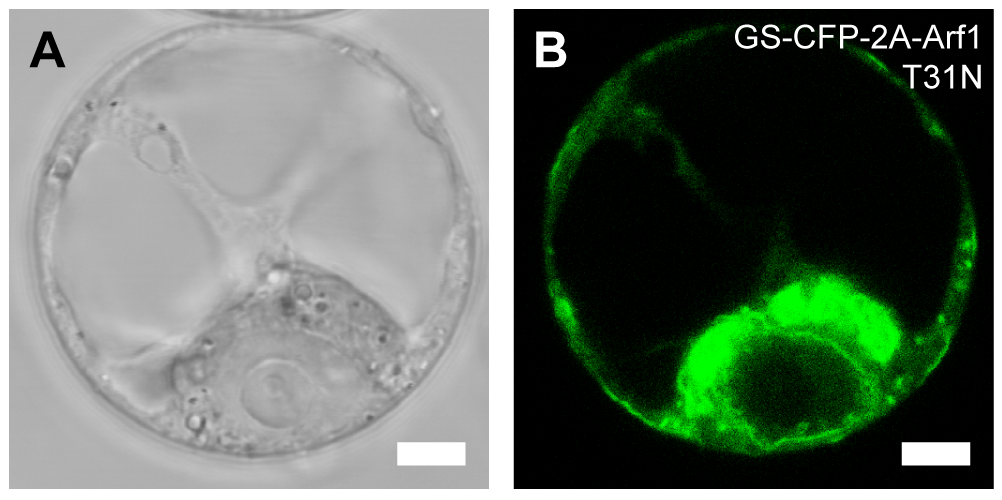

Supplement: Figure S7 — Accumulation of GS-CFP-2A in vacuole like structures when co-expressed with ARF1 (T31N). Bright field (A) and confocal (B) images of Arabidopsis protoplast transiently expressing GS-CFP-2A-ARF1 (T31N). In addition to the phenotype shown in Figure 5G–H, some protoplasts showed CFP fluorescence in vacuole like structures as shown here. Bars = 5 µm. (TIF) [file pone.0051973.s007.tif]

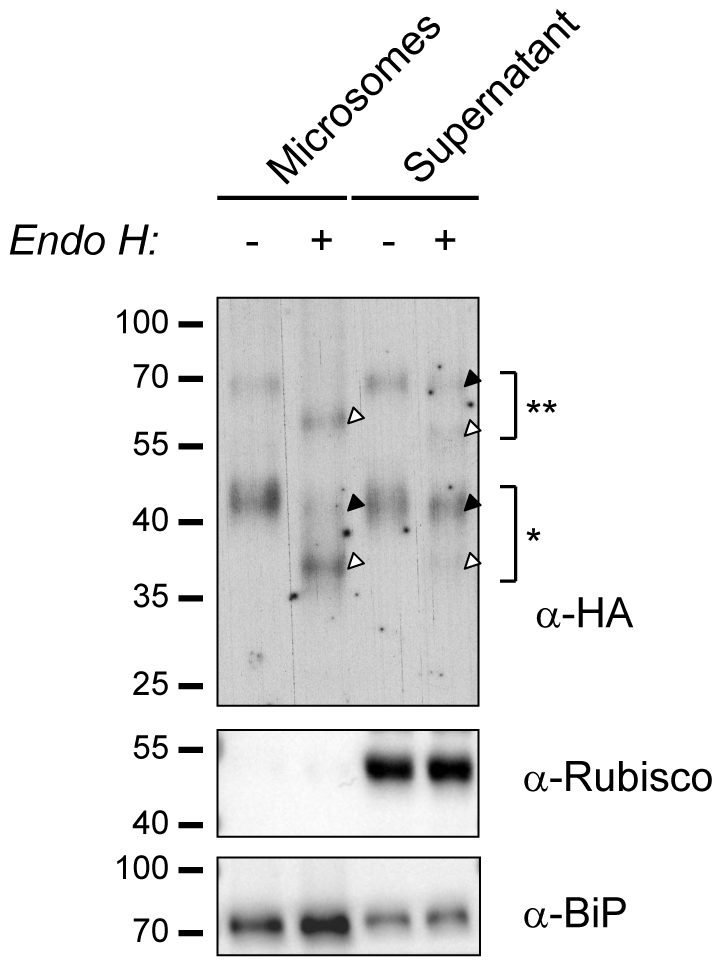

Supplement: Figure S8 — Endo H is a valid marker for protein trafficking. (A) Immunoblot analysis of ultracentrifuged protein extracts from Nicotiana benthamiana transiently expressing HACAH1-2A-RABD2a (wt) using anti-HA, anti-Rubisco and anti-BiP antibodies. Soluble stroma-containing and pelleted ER-containing microsome fractions are indicated. Samples were subjected (+) or not (−) to Endo H treatment. Non-cleaved full-length (**) and cleaved (*) products, as well as resistant (black triangle) and sensitive (white triangle) bands are indicated. (TIF) [file pone.0051973.s008.tif]

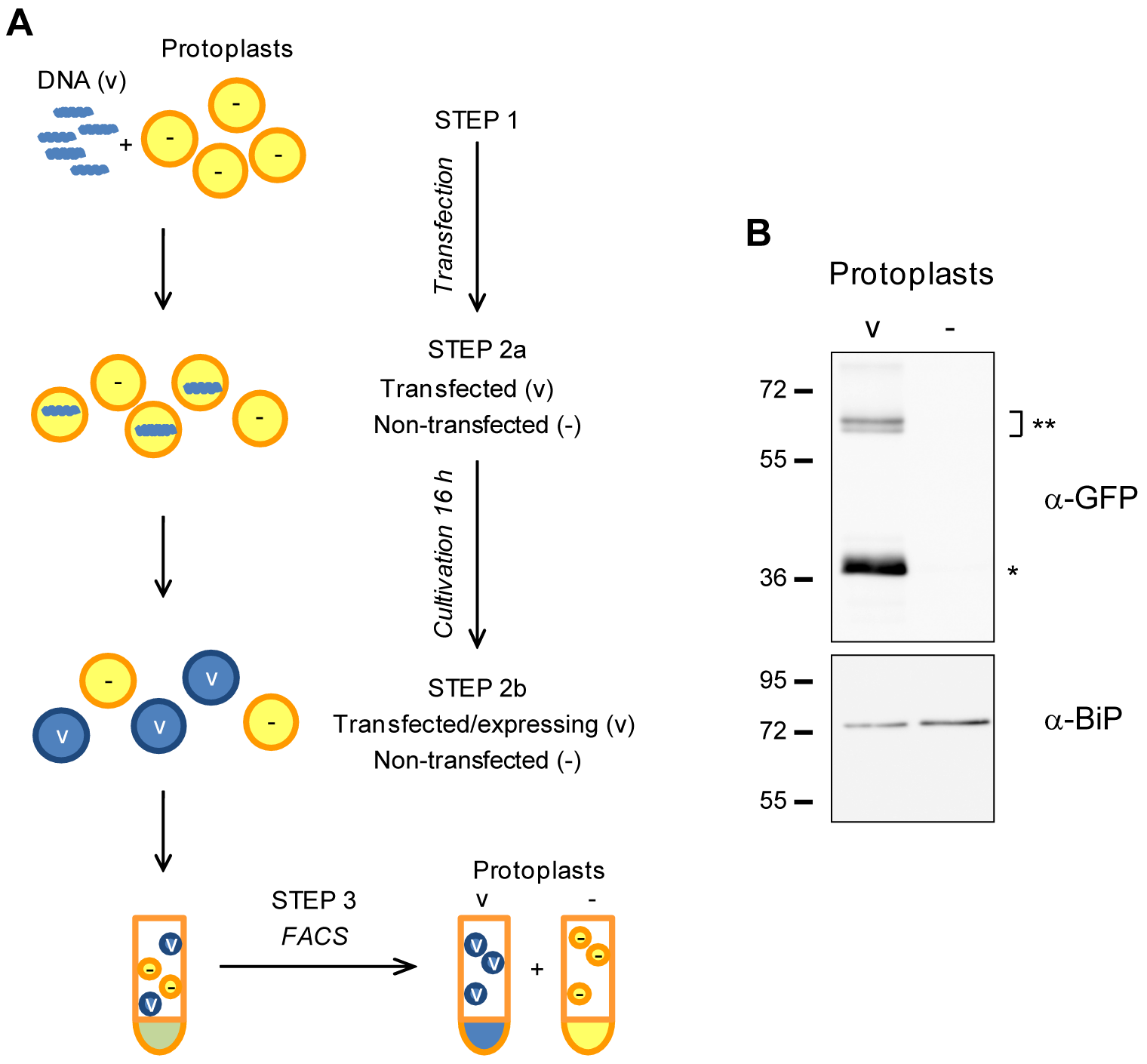

Supplement: Figure S9 — Sorting of 2A polyprotein transfected protoplasts using FACS. (A) Scheme of fluorescent-activated cell sorting (FACS) of protoplasts transfected with 2A polyprotein in order to obtain a homogenous mutant population. Protoplasts were transfected with GS-CFP-2A-RABD2a vector (v), resulting in a heterogeneous mixture of transfected (v, about 10% of total population, data not shown) or non-transfected cells (−). Protoplasts were sorted into two populations based on the level of CFP fluorescence (v or -, respectively). (B) Immunoblot analysis of total protoplast extracts from sorted cells transfected with GS-CFP-2A-RABD2a (v/−, transfected/non-transfected respectively) using anti-BiP (loading control) or anti-GFP antiserum. Non-cleaved GS-CFP-2A-RABD2a (**) and released GS-CFP-2A (*) are indicated. (TIF) [file pone.0051973.s009.tif]
