# Supplementary material for: Use of the Foot-and-Mouth Disease Virus 2A Peptide Co-Expression System to Study Intracellular Protein Trafficking in Arabidopsis
Source: PLoS One. 2012 Dec 14;7(12):e51973. doi: 10.1371/journal.pone.0051973 (PMC3522588; doi:10.1371/journal.pone.0051973)
Supplement: Table S1 — Intracellular distribution of HACAH1 in Col-0 Arabidopsis thaliana suspension cultured cells. Immunogold labeling density over several sub-cellular compartments was estimated in both wild type and HACAH1 stably transformed Col-0 Arabidopsis thaliana suspension cells using HA antiserum. (DOCX) [file pone.0051973.s010.docx]

| **Subcellular compartment** | **Immunogold density (gold particles/μm^2^ ± Standard deviation)** | |
| --- | --- | --- |
|  | **Wt** | **HACAH1** |
| Nucleus | 16.3 ± 9.8 | 16.3 ± 5.5 |
| ER | 22.0 ± 6.2 | 22.5 ± 7.2 |
| Golgi | 7.4 ± 6.7 | 13.2 ± 1.8 |
| Chloroplast | 6.6 ± 1.6 | 22.5 ± 5.4 |
| Vacuole | 0.8 ± 0.2 | 1.5 ± 0.6 |
| Cell Wall | 1.4 ± 0.5 | 2.2 ± 1.8 |
