# Supplementary material for: Use of the Foot-and-Mouth Disease Virus 2A Peptide Co-Expression System to Study Intracellular Protein Trafficking in Arabidopsis
Source: PLoS One. 2012 Dec 14;7(12):e51973. doi: 10.1371/journal.pone.0051973 (PMC3522588; doi:10.1371/journal.pone.0051973)
Supplement: Text S1 — Supporting Material and Methods. (DOCX) [file pone.0051973.s011.docx]

### *Inhibitor treatments of transiently transfected* Arabidopsis thaliana *protoplasts*

Five microliter BFA (Sigma), 5 mg/ml dissolved in ethanol, was added to 500 ul protoplasts solution 18 h post-transformation. Equal amounts of ethanol were added to control protoplasts. Protoplasts were analyzed 6 h after drug treatment (24 h post-transformation). CHX (Sigma), 10 mg/ml in 50% ethanol, was added to the protoplast media (final concentration of 100 µM) 6, 4 and 2 h before harvesting of protoplasts (21 h post-transformation).

### *Preparation of protein extracts and blot analysis*

For the fractionation studies, 1-2 leaves of agroinfiltrated *Nicotiana benthamiana* were grinded in 1 ml plant homogenization buffer (25 mM Tris-HCl pH 7.5, 250 mM sucrose, 2 mM MgCl_2_, 1 mM DTT) on ice and centrifuged at 13500 rpm for 20 min at 4 °C, The supernatant (total cellular fraction, TE) was then ultracentrifuged at 10000 g for 1h in a Beckman Coulter Optima L-100XP ultracentrifuge. The supernatant (stroma-containing fraction, SN) was collected and the pellet was resuspended in Buffer A (5 mM Tris-HCl pH 7.5, 0.25 mM sucrose, 2 mM Cl_2_Mg, 1 mM DTT) (microsome fraction, MS).

### *Immunogold and electron microscopy*

Immunogold and electron microscopy on *Arabidopsis* ecotype Columbia-0 cell cultures, wild-type and expressing HACAH1, was performed as previously described [1] with minor modifications. After the first antibody was added, grids were rinsed five times in TBS buffer containing 2 mg/mL BSA and three times in TBS buffer containing 0.1% (v/v) Triton X-100 and 2 mg/mL BSA. Last three brief bi-distilled water washings (3 min each) were performed before samples were allowed to dry at room temperature. Grids were post-stained for 5 min with uranyle acetate solution (to saturation in MetOH), washed with distilled water and incubated with Reynolds lead citrate for 45 s under a N_2_ atmosphere to avoid precipitation. After washing, dry sections were analysed in a JEOL JEM 1010 transmission electron microscope (Tokio, Japan), set to 80 KV. Images were captured using a BioScan camera (Gatan, Pleasanton, CA, USA). Gold particle density was defined as the average gold particle number per area unit. Organelle areas were calculated by using the UTHSCSA Image Tool software (3.0 version) according the manufacturers recommendations.

### *Flow Cytometry and FACS*

Transfected protoplasts were prepared for flow cytometry by filtering through a 41 µ nylon mesh. Total pre-sorted protoplast extract was prepared for SDS-PAGE. Filtered protoplast suspensions were cytometrically analyzed and sorted with a FACSAria (BD Biosciences) fitted with a 100-mm nozzle and using phosphate buffered saline as a sheath fluid [2]. A 488-nm Coherent Sapphire Solid State laser (emission measured at 530/30 nm, FITC) and a 407-nm Point Source Violet Solid State (emission measured at 530/30 nm, Alexa Fluor 430) was used for excitation. The photomultiplier tube voltage was set at 50 V for forward scatter, 392 V for side scatter, 467 V for FITC and 401 V for Alexa Fluor 430. The threshold value for event detection was set at 5,000 on forward scattering. The amplitude was set to 29.2 V and the drop delay value was 24.89. Data were processed using the FACSDiva 6.1.2 software (BD Biosciences). No compensation was used. Gates for sorting was defined by measuring CFP (Alexa Fluor 430 settings) and background fluorescence (FITC settings) using protoplasts transfected with GS-CFP-2A-RABD2a or empty vector. Gates were defined to allow 1% false positives in the negatively transfected population. This resulted in 13.2 % of the CFP transfected protoplasts being positively sorted, in good agreement with fluorescence microscopy based estimation of transformation efficiency. Positively and negatively sorted protoplasts were pelleted by low-speed centrifugation at 800 rpm for 5 min, followed by 2,500 rpm for 30 sec and prepared for SDS-PAGE.

**Supporting References**

1. Buren S, Ortega-Villasante C, Blanco-Rivero A, Martinez-Bernardini A, Shutova T, et al. (2011) Importance of post-translational modifications for functionality of a chloroplast-localized carbonic anhydrase (CAH1) in Arabidopsis thaliana. PLoS One 6: e21021.

2. Bargmann BO, Birnbaum KD (2009) Positive fluorescent selection permits precise, rapid, and in-depth overexpression analysis in plant protoplasts. Plant Physiol 149: 1231-1239.
